# Grafted Human iPS Cell-Derived Oligodendrocyte Precursor Cells Contribute to Robust Remyelination of Demyelinated Axons after Spinal Cord Injury

**DOI:** 10.1016/j.stemcr.2015.11.013

**Published:** 2015-12-24

**Authors:** Soya Kawabata, Morito Takano, Yuko Numasawa-Kuroiwa, Go Itakura, Yoshiomi Kobayashi, Yuichiro Nishiyama, Keiko Sugai, Soraya Nishimura, Hiroki Iwai, Miho Isoda, Shinsuke Shibata, Jun Kohyama, Akio Iwanami, Yoshiaki Toyama, Morio Matsumoto, Masaya Nakamura, Hideyuki Okano

**Affiliations:** 1 Department of Physiology, Keio University School of Medicine, 35 Shinanomachi, Shinjuku-ku, Tokyo 160-8582, Japan; 2 Department of Orthopaedic Surgery, Keio University School of Medicine, 35 Shinanomachi, Shinjuku-ku, Tokyo 160-8582, Japan; 3 Department of Pediatrics, Keio University School of Medicine, 35 Shinanomachi, Shinjuku-ku, Tokyo 160-8582, Japan

## Abstract

Murine- and human-induced pluripotent stem cell-derived neural stem/progenitor cells (iPSC-NS/PCs) promote functional recovery following transplantation into the injured spinal cord in rodents and primates. Although remyelination of spared demyelinated axons is a critical mechanism in the regeneration of the injured spinal cord, human iPSC-NS/PCs predominantly differentiate into neurons both in vitro and in vivo. We therefore took advantage of our recently developed protocol to obtain human-induced pluripotent stem cell-derived oligodendrocyte precursor cell-enriched neural stem/progenitor cells and report the benefits of transplanting these cells in a spinal cord injury (SCI) model. We describe how this approach contributes to the robust remyelination of demyelinated axons and facilitates functional recovery after SCI.

## Highlights

• hiPSC-OPC-enriched NS/PCs differentiate into mature oligodendrocytes in vivo
• Grafted hiPSC-OPCs-derived mature oligodendrocytes contribute to remyelination
• Transplanted hiPSC-OPC-enriched NS/PCs enhance functional recovery following SCI
• Transplanted hiPSC-OPC-enriched NS/PCs promote axonal growth of host neurons

## Graphical Abstract

**Figure:**
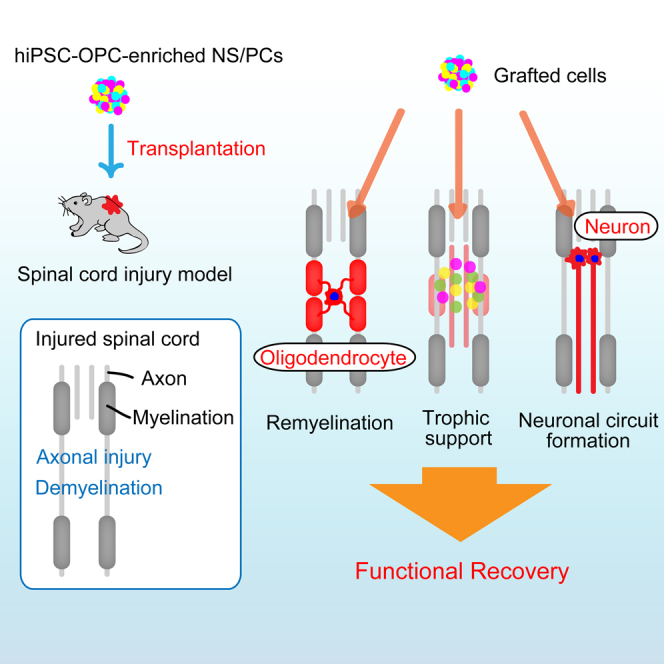

## Teaser

In this article, Okano and colleagues show that the benefits of transplanting human-induced pluripotent stem cell-derived oligodendrocyte precursor cell-enriched neural stem/progenitor cells, describing how this approach contributes to the robust remyelination of demyelinated axons and facilitates functional recovery after spinal cord injury.

## Introduction

Previous studies describing functional recovery following transplantation of neural stem/progenitor cells (NS/PCs) in spinal cord injury (SCI) models demonstrated the therapeutic promise of this approach (Cummings et al., 2005, Iwanami et al., 2005). A number of putative underlying mechanisms have been suggested, including cell replacement by grafted NS/PC-derived neurons, astrocytes, and oligodendrocytes; trophic support for increased survival of the host neural cells and host-mediated repair processes; and, more recently, axonal remyelination by grafted NS/PC-derived oligodendrocytes (Cummings et al., 2005, Keirstead et al., 2005, Salewski et al., 2015, Yasuda et al., 2011). Human NS/PCs and human-induced pluripotent stem cell-derived NS/PCs (hiPSC-NS/PCs) predominantly differentiate into neurons, and, to a lesser extent, into mature oligodendrocytes both in vitro and in vivo (Kobayashi et al., 2012, Nori et al., 2011, Nori et al., 2015, Romanyuk et al., 2015). We therefore developed a protocol for the induction of oligodendroglial differentiation of hiPSC-NS/PCs in vitro (Numasawa-Kuroiwa et al., 2014). In the present study, we used a pre-evaluated safe line of induced pluripotent stem cells (iPSCs; 201B7) (Nori et al., 2011, Nori et al., 2015) and induced their differentiation into oligodendrocyte precursor cell-enriched NS/PCs (hiPSC-OPC-enriched NS/PCs). The aim of this study was to evaluate the therapeutic potential of hiPSC-OPC-enriched NS/PCs in the treatment of SCI.

## Results

### hiPSC-OPC-Enriched NS/PCs Differentiate into Mature Oligodendrocytes In Vitro and In Vivo

**Figure 1:**
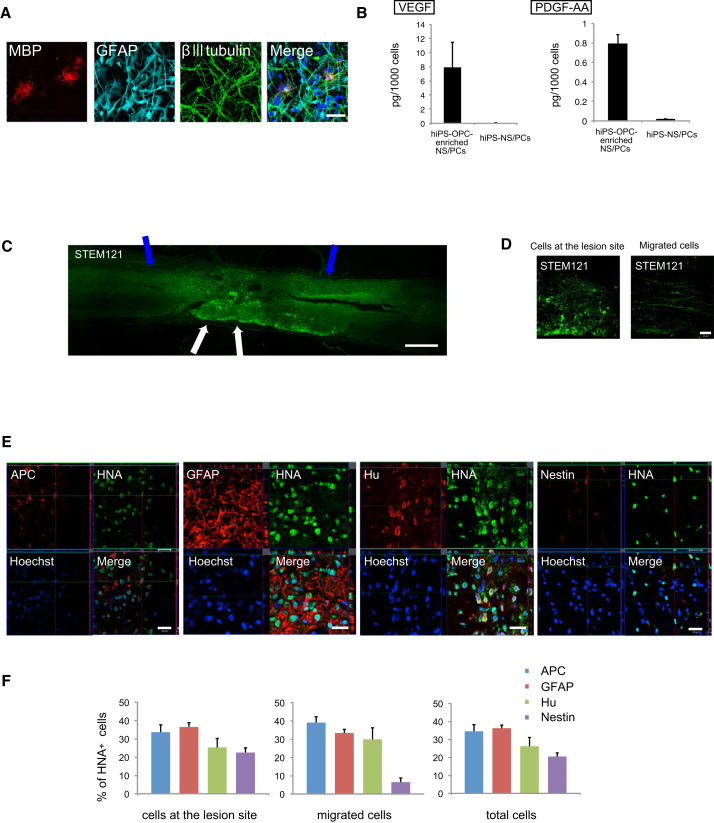
Differentiation Potential of hiPSC-OPC-Enriched NS/PCs In Vitro and In Vivo (A) Representative immunocytochemical images of three neural lineage cells (neurons, βIII tubulin: astrocytes, GFAP; oligodendrocytes, MBP). Scale bar, 100 μm. (B) Quantification of secreted cytokines from hiPSC-OPC-enriched NS/PCs and hiPSC-NS/PCs. Secretion of VEGF and PDGF-AA was significantly higher in hiPSC-OPC-enriched NS/PC cultures than in hiPSC-NS/PC cultures (n = 3 independent experiments). (C) STEM121^+^ grafted cells were integrated at the lesion epicenter (white arrows) and migrated rostrally and caudally (blue arrows). Scale bar, 500 μm. (D) Representative images of STEM121^+^ grafted cells at the lesion site and distant areas. Scale bar, 50 μm. (E) Representative images of HNA^+^ grafted cells together with APC^+^ oligodendrocytes, GFAP^+^ astrocytes, Hu^+^ neurons, and Nestin^+^ immature cells. Scale bar, 20 μm. (F) Percentages of cell-type-specific marker-positive cells among HNA^+^-grafted cells at the lesion site, at distant areas, and in total cells (n = 5 independent experiments). See also Figures S1 and S2.

We induced hiPSC-OPC-enriched NS/PCs from a pre-evaluated safe iPSC line (201B7) (Nori et al., 2011) following a previously reported protocol (Numasawa-Kuroiwa et al., 2014) (Figure S1). Immunocytochemical analyses revealed that hiPSC-OPC-enriched NS/PCs differentiated into myelin basic protein (MBP)^+^ mature oligodendrocytes in vitro. This differentiation was not observed using conventional hiPSC-NS/PCs prepared following previously reported protocols (Nori et al., 2011). hiPSC-OPC-enriched NS/PCs also differentiated into β-tubulin isotype III (βIII tubulin)^+^ neurons and glial fibrillary acidic protein (GFAP)^+^ astrocytes (Figure 1A). Furthermore, the cytokine expression profile list obtained using a cytokine antibody array showed that significantly higher levels of vascular endothelial growth factor (VEGF) and platelet-derived growth factor (PDGF)-AA were secreted into the culture medium of hiPSC-OPC-enriched NS/PCs than into that of conventional hiPSC-NS/PCs (Figure 1B). Other cytokines such as β-nerve growth factor, brain-derived neurotrophic factor, ciliary neurotrophic factor, glial cell-derived neurotrophic factor, hepatocyte growth factor, neurotrophin-3, and neurotrophin-4 were not detected in either group.

Contusive SCI was induced at the Th10 level in NOD-SCID mice, and 5 × 10^5^ hiPSC-OPC-enriched NS/PCs were transplanted into the lesion epicenter 9 days later. Twelve weeks after transplantation, we performed immunohistochemical analyses using antibodies specific to human cytoplasm (STEM121) (Cummings et al., 2005), human nuclear antigen (HNA), and cell-type-specific markers. Engrafted hiPSC-OPC-enriched NS/PCs differentiated into adenomatous polyposis coli CC-1 (APC)^+^ mature oligodendrocytes, Hu^+^ neurons, and GFAP^+^ astrocytes (Figure 1D). Some transplanted cells were present at the lesion site, and the others migrated diffusely into the host spinal cord (Figure 1C). We quantified the proportion of HNA^+^ cells immunopositive for cell-type-specific markers in each site. Of the migrated cells, 39.2% ± 3.1% had differentiated into APC^+^/HNA^+^ mature oligodendrocytes, 33.3% ± 2.0% into GFAP^+^/HNA^+^ astrocytes, 29.9% ± 6.4% into Hu^+^/HNA^+^ neurons, and 6.34% ± 2.5% into Nestin^+^/HNA^+^ cells. At the lesion site, 33.6% ± 4.1% of HNA^+^ cells were APC^+^/HNA^+^ mature oligodendrocytes, 36.6% ± 2.1% were GFAP^+^/HNA^+^ astrocytes, 25.3% ± 4.8% were Hu^+^/HNA^+^ neurons, and 22.6% ± 2.5% were Nestin^+^/HNA^+^ cells. Of the total population of differentiated cells within the host spinal cord, 34.5% ± 3.7% were APC^+^ cells, 36.1% ± 1.8% were GFAP^+^ cells, 26.1% ± 5.0% were Hu^+^ cells, and 20.4% ± 2.2% were Nestin^+^ cells (Figure 1E).

### Grafted hiPSC-OPC-Derived Mature Oligodendrocytes Contribute to Remyelination

**Figure 2:**
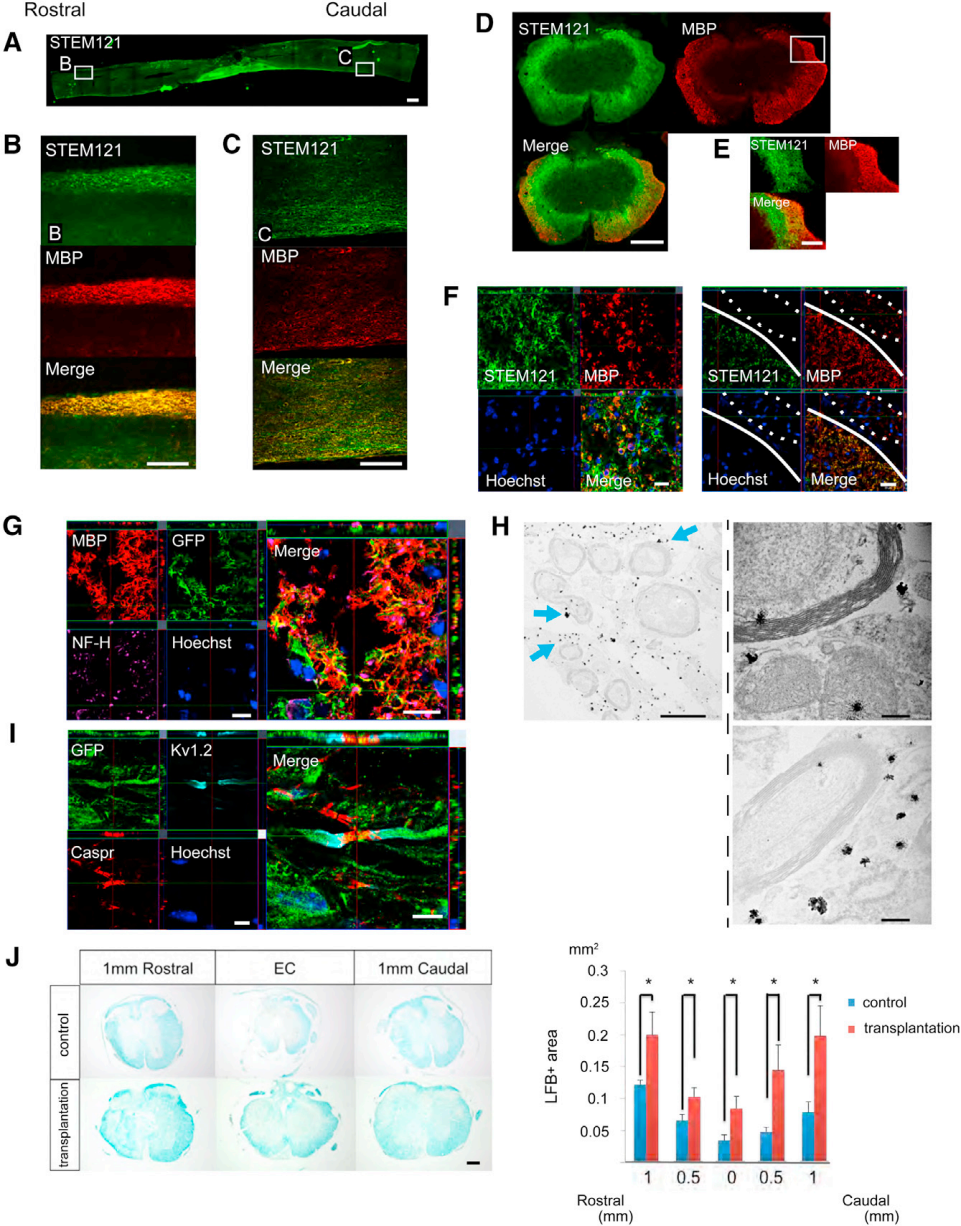
Grafted hiPSC-OPC-Derived Oligodendrocytes Contribute to Remyelination of Spared Axons (A–C) Representative images of sagittal sections stained for STEM121 and MBP. STEM121^+^/MBP^+^ hiPSC-OPC-derived mature oligodendrocytes migrated rostrally and caudally. Scale bar, 500 μm in (A), 300 μm in (B) and (C). (D–F) Representative images of stained axial sections. hiPSC-OPC-derived oligodendrocytes migrated into white matter broadly. The region enclosed by the dashed line indicates the dorsal root. Scale bar, 300 μm in (D), 100 μm in (E), and 20 μm in (F). (G) Representative images of axial sections stained for MBP, GFP, and NF-H. Many GFP^+^/MBP^+^ double-labeled myelin sheaths were observed around NF-H^+^ neuronal fibers. Scale bar, 10 μm. (H) Representative images of immunoelectron microscopy. Grafted cells were detectable by the black dots observed upon anti-GFP antibody staining. Anti-GFP antibody labeling was often observed in the outer cytoplasm of the myelin sheath (blue arrows). At a high magnification, remyelinated axons surrounded by GFP^+^ transplanted cells were identified. Scale bar, 2 μm (left), 200 nm (right). (I) Representative images of sagittal sections stained for GFP, Caspr, and Kv1.2. Nodes of Ranvier, identified with antibodies for the paranodal Caspr protein and the juxtaparanodal voltage-gated potassium channel protein Kv1.2, were observed in GFP^+^ myelin sheathes. Scale bar, 10 μm. (J) Representative images of LFB staining at the lesion epicenter and at sites 1-mm rostral and caudal. Scale bar, 200 μm. Quantitative analyses revealed a significantly larger myelinated area in the transplantation group than in the control group. Values are means ± SEM (control group, n = 6; transplantation group, n = 5; ^∗^p < 0.05). See also Figure S3.

A number of STEM121^+^/MBP^+^ double-positive areas, i.e., areas including human oligodendrocytes, were observed in white matter of the injured spinal cord (Figures 2A–2C), suggesting that many engrafted cells had differentiated into mature oligodendrocytes, forming thick sheathes and migrating diffusely into white matter, but not into gray matter, of the injured spinal cord (Figures 2D–2F). We labeled transplanted cells with Venus fluorescent protein engineered from the original GFP (Nagai et al., 2002). Immunohistochemical analyses revealed large amounts of GFP^+^/MBP^+^ double-labeled myelin sheaths surrounding NF-H^+^ axons (Figure 2G), suggesting that graft-derived human oligodendrocytes form myelin sheaths. Immunoelectron microscopy showed that immature myelin sheaths were strongly associated with myelin cytoplasm with nanogold-labeled GFP^+^ spots (Figure 2H). These results indicate that transplanted hiPSC-OPC-derived oligodendrocytes form mature myelin sheaths on spared axons. In addition, nodes of Ranvier, identified with antibodies to the paranodal Caspr protein (Rios et al., 2000) and the juxtaparanodal voltage-gated potassium channel protein Kv1.2 (Wang et al., 1993), were observed in transplanted cell-derived myelin sheaths (Figure 2I). Luxol Fast Blue (LFB) staining showed significant differences between the transplantation and control groups in LFB^+^ myelinated areas at all sites examined (Figure 2J). Furthermore, immunohistochemistry revealed that MBP^+^ areas in white matter were significantly larger in the transplantation group than in the control group (Figure S3).

### Transplanted hiPSC-OPC-Enriched NS/PCs Promote Axonal Growth and Contribute to Synapse Formation between Graft Cell-Derived Neurons and Host Mouse Neurons

**Figure 3:**
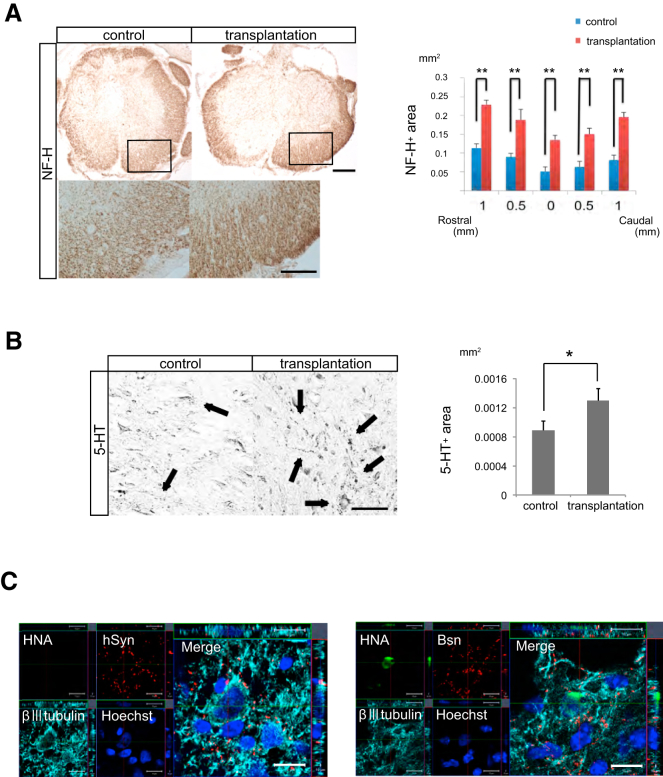
Transplanted hiPSC-OPC-Enriched NS/PCs Promote Axonal Growth and Contribute to Synapse Formation between Grafted Cell-Derived Neurons and Host Mouse Neurons (A) Representative images of NF-H^+^ neurofilament staining. Scale bar, 200 μm (upper) and 20 μm (lower). Quantification of NF-H^+^ areas revealed that neuronal fiber areas were significantly larger in the transplantation group than in the control group. Values are means ± SEM (n = 5, ^∗∗^p <0.01). (B) Representative images of axial sections stained for 5-HT at 4-mm caudal to the epicenter. Black arrows indicate 5-HT^+^ fibers. Scale bar, 50 μm. Quantitative analyses revealed that there were significantly more 5-HT^+^ fibers in the transplantation group than in the control group. Values are means ± SEM (control group, n = 6; transplantation group, n = 5; ^∗^p < 0.05). (C) Representative images showing staining for HNA, βIII tubulin, and the human-specific presynaptic marker hSyn or the mouse presynaptic marker Bsn. hSyn^+^ boutons were apposed to βIII tubulin^+^/HNA^−^ host mouse neurons (left). In addition, βIII tubulin^+^/HNA^+^ grafted cell-derived neurons were observed in contact with Bsn^+^ cells. Scale bar, 10 μm.

We examined the effects of transplanted cells on axonal growth after SCI by immunohistochemical analyses using antibodies for anti-NF-H (a marker of large-diameter neurofilaments) and anti-5-hydroxytryptamine (5-HT; a marker of raphespinal serotonergic fibers). NF-H^+^ neuronal fibers were prominent in the transplantation group compared with the control group. There were statistically significant differences in NF-H^+^ areas at all sites examined between the two groups (Figure 3A). At the lumbar enlargement, more 5-HT^+^ serotonergic fibers were observed in the transplantation group than in the control group (Figure 3B).

To assess the ability of transplanted cell-derived neurons to integrate with the host neural circuitry, we performed triple immunostaining with antibodies to HNA, βIII tubulin, and mouse-specific Bassoon (Bsn), a presynaptic marker. βIII tubulin^+^/HNA^+^ graft-derived neurons co-localized with Bsn^+^ synaptic boutons of host neurons (Figure 3C). Triple immunostaining for HNA, βIII tubulin, and human-specific synaptophysin (hSyn) revealed that hSyn^+^ synaptic boutons were apposed to βIII tubulin^+^/HNA^−^ host mouse neurons (Figure 3C). These findings suggest that graft-derived neurons integrate with host neuronal circuits and form synapses.

### Transplanted hiPSC-OPC-Enriched NS/PCs Enhance Functional Recovery Following SCI

**Figure 4:**
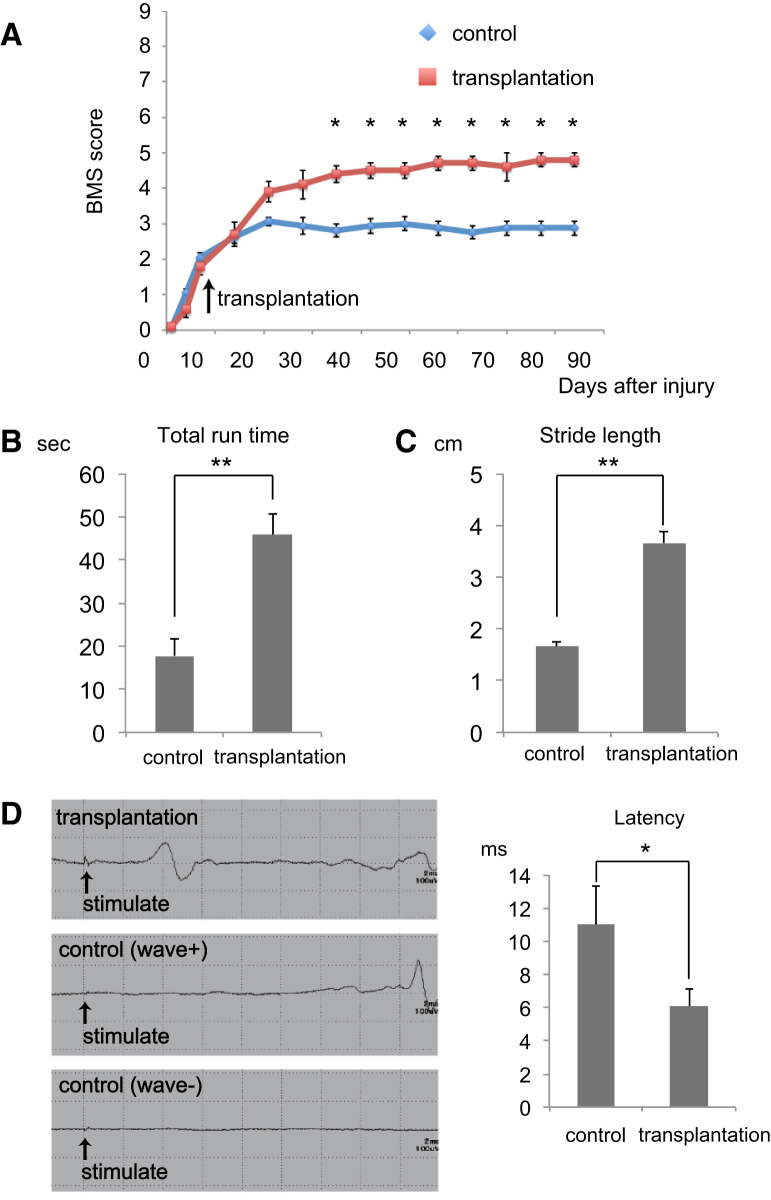
Transplantation of hiPSC-OPC-enriched NS/PCs Promotes Motor Functional and Electrophysiological Recovery after SCI (A) The BMS score showed that functional recovery was significantly better in the transplantation group than in the control group at 35 days after SCI and thereafter. Values are means ± SEM (control group, n = 8; transplantation group, n = 5; ^∗^p < 0.05). (B) Time on the rotating rod in each group at 12 weeks after transplantation. Transplanted mice could run on the rod for significantly longer than control mice. Values are means ± SEM (control group, n = 8; transplantation group, n = 5; ^∗∗^p < 0.01). (C) Treadmill gait analyses using the DigiGait System at 12 weeks after transplantation. Stride length was significantly longer in the transplantation group than in the control group. Values are means ± SEM (control group, n = 8; transplantation group, n = 5; ^∗∗^p < 0.01). (D) Electrophysiological analyses at 12 weeks after transplantation. Although MEP waves were detected in all transplanted mice, no waves were detected in 50% of control mice. The graph shows the latency of the MEP in transplanted mice and control mice in which waves were observed. The latency was significantly shorter in the transplantation group than in the control group. Values are means ± SEM (control group, n = 5; transplantation group, n = 5; ^∗^p < 0.05).

Recovery of motor function was assessed by the Basso Mouse Scale (BMS), Rota-rod test, and DigiGait analysis. The BMS score indicated significantly improved motor function in the transplantation group compared with the control group at 35 days post-SCI and beyond (Figure 4A). In the Rota-rod treadmill test, transplanted mice were able to run for significantly longer than control mice at 12 weeks after transplantation (Figure 4B). Using the DigiGait Image Analysis System to analyze gait, we found that stride was significantly longer in the transplantation group than in the control group at 12 weeks after transplantation (Figure 4C).

Because remyelination facilitates the restoration of rapid nerve conduction, we performed electrophysiological examination using the motor-evoked potential (MEP) at 12 weeks post-transplantation. The latency of the MEP in each wave was measured starting from the time of the stimulus. Although we detected an MEP wave in all transplanted mice, we detected MEP waves in only 50% of control mice (Figure 4D). MEP latency was significantly longer in the control group than in the transplantation group (Figure 4D), suggesting that graft-derived oligodendrocytes contribute to an increase in myelinated fibers.

## Discussion

### Transplanted hiPSC-OPC-Derived Oligodendrocytes Contribute to Remyelination of Spared Axons

The 201B7 hiPSC line is considered to be a safe clone based on previous studies showing the absence of observed tumorigenic propensities upon transplantation into the SCI model of NOD/SCID mice (Nori et al., 2015). In the present study, we used these safe iPSCs and prepared hiPSC-derived OPC-enriched NS/PCs as a cell source to treat SCI in adult NOD-SCID mice. Tumor formation was not observed at 12 weeks after transplantation.

Transplantation of hiPSC-OPC-enriched NS/PCs promoted functional recovery after SCI via differentiation into neurons, astrocytes, and mature oligodendrocytes. Using conventional hiPSC-NS/PCs, approximately 50% of grafted cells differentiate into neurons and only 3% differentiate into APC^+^ oligodendrocytes (Nori et al., 2011), while 35% of hiPSC-OPC-enriched NS/PCs differentiate into mature oligodendrocytes. Moreover, immunohistochemistry and electron microscopy revealed direct remyelination of spared axons.

Post-SCI inflammation has both beneficial and deleterious effects on transplanted cells. Inflammation may promote the survival and migration of transplanted cells by inducing the secretion of trophic factors (Copelman et al., 2000, Diemel et al., 2003, Hinks and Franklin, 1999); however, phagocytosis and the release of pro-inflammatory cytokines by activated macrophages and microglia can harm engrafted cells (Bartnik et al., 2000). One feature of OPCs is their ability to migrate into demyelinated lesions (Piaton et al., 2011). Previous studies using rat models of multiple sclerosis showed that OPCs migrate toward inflamed demyelinated lesions (Wang et al., 2011), suggesting that inflammation affects migration and interactions between OPCs and host tissue. Consistent with this, we found that transplanted hiPSC-OPCs migrate broadly into host white matter and contribute extensively to remyelination of demyelinated axons. Such effects may contribute to the improvement of locomotor function.

### Graft Cell-Derived Neurons Contribute to Neuronal Relay by Synapse Formation with Host Mouse Neurons

Previous studies demonstrated post-SCI functional improvement associated with grafted neuronal progenitor cells (Abematsu et al., 2010, Bonner et al., 2010, Cummings et al., 2005). Abematsu et al. (2010) reported that ablation of transplanted iPSC-NS/PCs leads to loss of motor function, suggesting that the survival of graft-derived cells is essential to maintain function. They also showed that graft-derived human neurons receive projections from host mouse neurons, and that their extended processes form synapses with host neurons. In this study, grafted hiPSC-OPC-enriched NS/PCs differentiated into both oligodendrocytes and neurons capable of forming synapses with host neurons within the injured spinal cord, suggesting that the reconstruction of neuronal circuits also plays a substantial role in functional recovery after transplantation of hiPSC-OPC-enriched NS/PCs. All et al. (2015) reported the effectiveness of hiPSC-derived OPC transplantation for SCI in rats. However, their cells predominantly differentiate into oligodendrocytes, and less so into neurons. Graft-derived neurons are also expected to play a significant role in improving functional recovery after transplantation.

We did not transplant conventional hiPSC-NS/PCs in this study because we reported solid data about the transplantation of conventional hiPSC-NS/PCs into an SCI model in NOD/SCID mice (prepared under the same conditions as in the present study) in our previous studies (Nori et al., 2011, Nori et al., 2015). Thus, we simply compared the behavioral data of two groups (animals grafted with hiPSC-OPC-enriched NS/PCs in the present study and those grafted with conventional hiPSC-NS/PCs in previous studies). Although the therapeutic potential of hiPSC-OPC-enriched NS/PCs should in theory differ from that of conventional hiPSC-NS/PCs, we did not find any differences in the BMS score, the Rota-rod treadmill test, or the DigiGait Image Analysis System, which may reflect difficulties in assessing motor function in mice and in differentiating between the two groups. Further studies using larger animal SCI models, such as common marmosets, which will enable us to evaluate skilled movements, may be needed to obtain significant differences in motor function recovery between hiPSC-OPC-enriched NS/PC and hiPSC-NS/PC transplantation.

### Grafted Cell-Derived Trophic Factors Enhance the Survival of Host Neural Cells and Host-Mediated Regeneration and Repair without Tumorigenicity

Axonal regrowth supported by astrocytes derived from transplanted cells may be another reason for the observed recovery. A previous report indicated that graft cell-derived immature astrocytes in injured spinal cords promote the outgrowth of 5-HT^+^ fibers by providing a growth-permissive surface (Hofstetter et al., 2002). Consistent with this, transplantation of hiPSC-OPC-enriched NS/PCs promoted serotonergic innervation of the distal cord compared with vehicle control mice, and the release of trophic factors by undifferentiated NS/PCs may protect against neural cell death, and, hence, glial scar formation (Brock et al., 2010, Tuszynski et al., 1996). Here, we show that hiPSC-OPC-enriched NS/PCs secrete several growth factors in vitro, enhance the sparing and/or regrowth of NF-H^+^ neuronal fibers, and prevent atrophy of the injured spinal cord.

In conclusion, OPC-enriched NS/PCs derived from the human iPSC clone 201B7 survived and differentiated into three neural lineage cells in the injured spinal cord of adult mice, with no evidence of tumor formation. Oligodendrocytes derived from transplanted cells may thus play a key role in promoting functional recovery by remyelinating spared axons.

## Experimental Procedures

### Cell Culture, Neural Induction, and Lentivirus Transduction

Culture and neural induction of hiPSCs (201B7) were performed as described previously (Numasawa-Kuroiwa et al., 2014). Lentivirus was prepared and transduced into neurospheres according to previously described methods (Okada et al., 2008). Briefly, hiPSC-derived primary neurospheres were dissociated and infected with lentivirus-expressing Venus fluorescent protein under the control of the elongation factor promoter (pCSII-EF-Venus). These primary neurospheres were passaged into secondary neurospheres and used for transplantation.

### SCI Model and Transplantation

Contusive SCI was induced at the Th10 level in spinal cords of adult female NOD-SCID mice. Nine days after injury, 5 × 10^5^ hiPSC-OPC-enriched NS/PCs were transplanted into the lesion epicenter of each mouse. All experiments were approved by the ethics committee of Keio University. Detailed methods are described in the Supplemental Information.

### Motor Function Analyses

Hindlimb motor function was evaluated for 12 weeks after transplantation using the BMS (Basso et al., 2006), a rotating rod apparatus, and the DigiGait System (Mouse Specifics). Detailed methods are described in the Supplemental Information.

### Histological Analyses

Histological analyses were performed at 12 weeks after transplantation. Sections were evaluated by H&E staining, LFB staining, and immunohistochemistry. Detailed protocols are described in the Supplemental Information.

### Statistical Analyses

All data are reported as the mean ± SEM. For all histological examinations, an unpaired two-tailed Student's t-test was used for single comparisons between the transplantation and vehicle control groups. The BMS scores were analyzed using the Mann-Whitney U-test. In each case, ^∗^p < 0.05 and ^∗∗^p < 0.01 were considered to be statistically significant.

## Supplemental Information

Document S1. Figures S1–S3 and Supplemental Experimental Procedures

Document S2. Article plus Supplemental Information
