# Supplementary material for: Grafted Human iPS Cell-Derived Oligodendrocyte Precursor Cells Contribute to Robust Remyelination of Demyelinated Axons after Spinal Cord Injury
Source: Stem Cell Reports. 2015 Dec 24;6(1):1–8. doi: 10.1016/j.stemcr.2015.11.013 (PMC4719132; doi:10.1016/j.stemcr.2015.11.013)
Supplement: Document S1. Figures S1–S3 and Supplemental Experimental Procedures [file mmc1.pdf]

Stem Cell Reports, Volume *6*

Supplemental Information

Grafted Human iPS Cell-Derived Oligodendrocyte Precursor Cells Contribute to Robust Remyelination of Demyelinated Axons after Spinal Cord Injury

Soya Kawabata, Morito Takano, Yuko Numasawa-Kuroiwa, Go Itakura, Yoshiomi Kobayashi, Yuichiro Nishiyama, Keiko Sugai, Soraya Nishimura, Hiroki Iwai, Miho Isoda, Shinsuke Shibata, Jun Kohyama, Akio Iwanami, Yoshiaki Toyama, Morio Matsumoto, Masaya Nakamura, and Hideyuki Okano

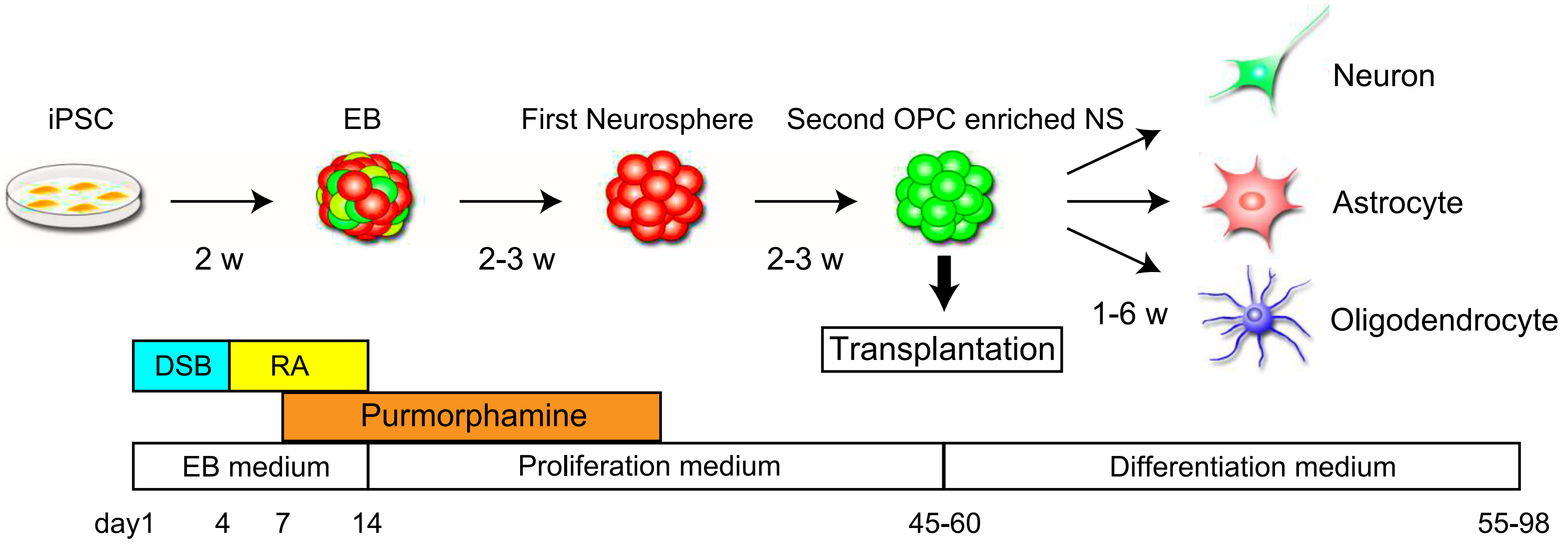
iPSC
EB
First Neurosphere
Second OPC enriched NS
Neuron
Astrocyte
Oligodendrocyte
2 w
2-3 w
2-3 w
1-6 w
Transplantation
DSB
RA
Purmorphamine
EB medium
Proliferation medium
Differentiation medium
day1
4
7
14
45-60
55-98

Figure S1

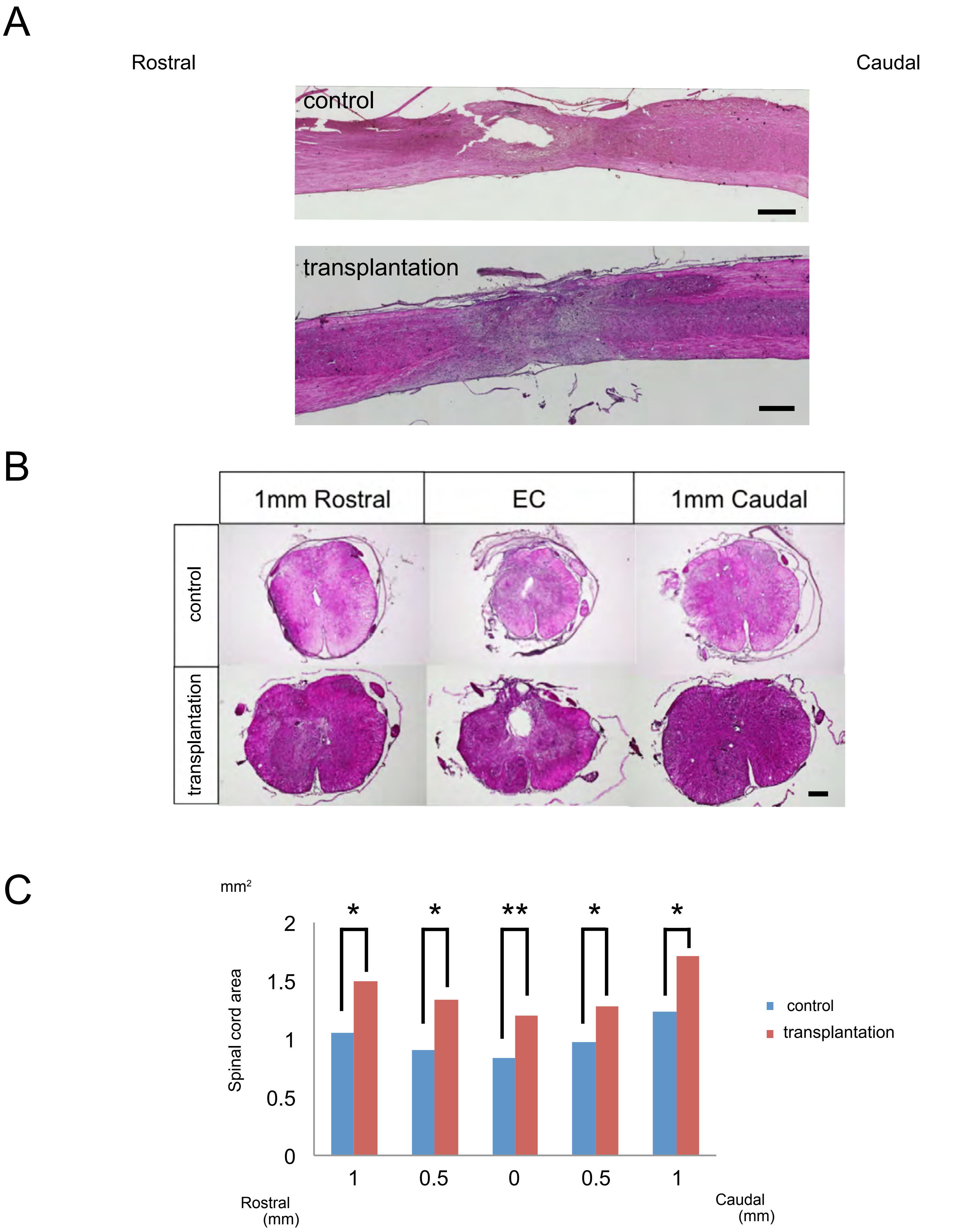
A
Rostral
Caudal
control
transplantation
B
1mm Rostral
EC
1mm Caudal
control
transplantation
C
mm2
Spinal cord area
2
1.5
1
0.5
0
*
*
**
*
*
1
0.5
0
0.5
1
Rostral (mm)
Caudal (mm)
control
transplantation

Figure S2

A

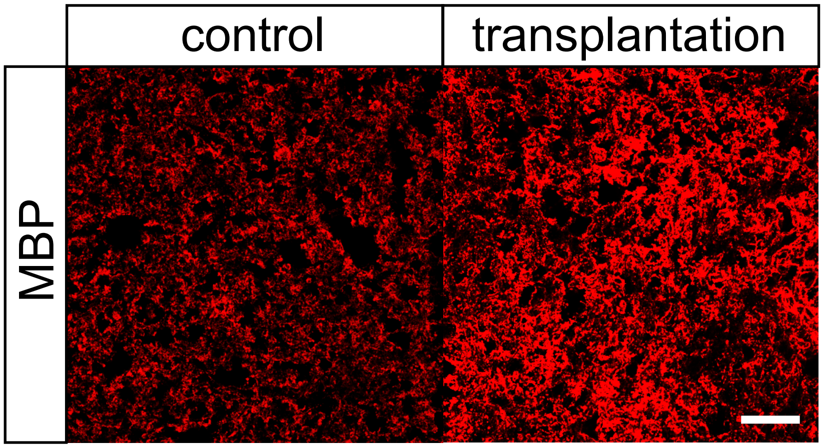
control
transplantation
MBP

B

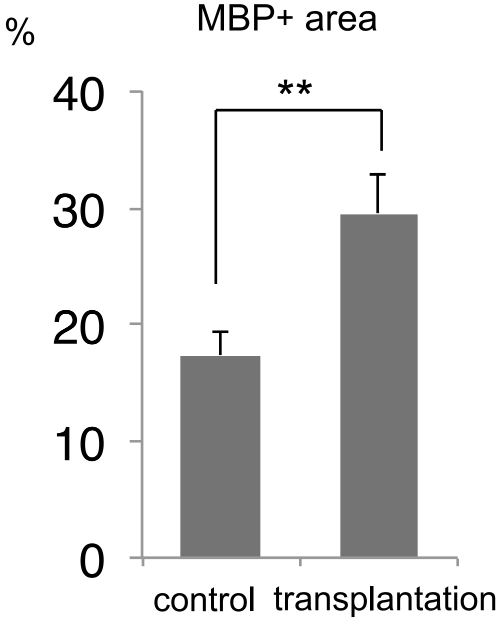
%
MBP+ area
40
30
20
10
0
**
control
transplantation

Figure S3

Supplemental Information

Figure Legends

Figure S1. Differentiation protocols

Schematic presentation of the protocols for hiPSC-OPC-enriched NS/PCs. DSB, dorsomorphin (D), SB431542 (S), and BIO (B); RA, retinoic acid; EB, embryoid body.

Figure S2. Transplantation of hiPSC-OPC-enriched NS/PCs prevents atrophy of the injured spinal cord without any tumorigenicity

A, B. Representative images of hematoxylin-eosin staining of sagittal and axial sections at 12 weeks after transplantation. Tumor formation was not observed in any mice at 12 weeks after transplantation. Scale bar, 500 μm (upper) and 200 μm (lower).

C. Quantitative analysis of spinal cord areas revealed that transverse areas of the injured spinal cord were significantly larger in the transplantation group than in the control group. Values are means ± SEM (control group, n = 6; transplantation group, n = 5; P* < 0.05; P**<0.01).

Figure S3. Transplantation of hiPSC-OPC-enriched NS/PCs enlarges MBP^+ areas in white matter

A. Representative images of axial sections stained for MBP. Scale bar, 20 μm.

B. MBP^+ percentage areas in white matter. Quantitative analyses revealed that the MBP^+ area was significantly larger in the transplantation group than in the control group. Values are means ± SEM (n=5, P** < 0.01).

Supplemental Experimental Procedures

Cytokine array

To obtain conditioned medium, hiPSC-OPC-enriched NS/PCs and conventional NS/PCs were cultured separately in growth medium. At 48 hours before collection, cells were washed once with proliferation medium consisting of serum-free medium and the medium was replaced with fresh growth medium. For background subtraction, the same amount of growth medium without cells was also incubated. The culture media were collected after 48 hours, centrifuged to remove cell debris, and stored at −80°C. The number of viable cells in each culture was calculated for the normalization. Cytokine profiles were obtained using human quantitative custom antibody arrays (RayBiotech, Norcross, GA, USA) to detect nine cytokines, namely, VEGF, PDGF-AA, β-nerve growth factor, brain-derived neurotrophic factor, ciliary neurotrophic factor, glial cell-derived neurotrophic factor, hepatocyte growth factor, neurotrophin-3, and neurotrophin-4, which were measured and processed according to the manufacturer's recommendation. The antibody array was a glass chip-based multiplexed sandwich enzyme-linked immunosorbent assay system. A standard glass slide was spotted with 16 wells of identical biomarker antibody arrays. Each antibody, together with the positive and negative controls, was arrayed in quadruplicate. The samples and standards were added to the wells of the chip array and incubated overnight at 4ºC. This was followed by five washing steps and the addition of a biotinylated antibody and Cy3 equivalent dye labeled-streptavidin to the wells. The signals were scanned and extracted with InnoScan 710 and Mapix software (Innopsys, Carbonne, France). After background subtraction, concentrations were calculated against a standard curve for each biomarker generated from the positive

and negative controls using Quantibody Analyzer software (RayBiotech, Norcross, GA, USA). Cytokine release into culture supernatants was normalized according to the number of viable cells in each culture.

SCI model and transplantation

Adult female NOD-SCID mice (20–22 g) were anesthetized with an intraperitoneal injection of ketamine (100 mg/kg) and xylazine (10 mg/kg). Contusive SCI was induced at the Th10 level using an IH impactor (60 kdyn; Precision Systems and Instrumentation, Lexington, USA), as described previously (Scheff et al., 2003). Nine days after injury, 5×10^5 hiPSC-OPC-enriched NS/PCs were transplanted into the lesion epicenter of each mouse using a glass pipette and a microstereotaxic injection system (KDS310; Muromachi-Kikai Co., Ltd., Tokyo, Japan). An equal volume of phosphate-buffered saline was injected into the lesion site for vehicle control mice.

Motor function analyses

Hind limb motor function was evaluated for 12 weeks after transplantation using the BMS (Basso et al., 2006). Well-trained investigators, blinded to the treatments, performed the behavioral analyses. Motor function was also measured on a rotating rod apparatus (KDS310; Muromachi-Kikai Co., Ltd., Tokyo, Japan), which consisted of a plastic rod (3 cm diameter) with a gritted surface. At 12 weeks after transplantation, each mouse was tested by monitoring the amount of time spent on the rod as it was rotated at 20 rpm. Gait analysis was performed using the DigiGait System (Mouse Specifics, Quincy, MA, USA) at 12 weeks after transplantation.

Histological analyses

All mice were deeply anesthetized and transcardially perfused with 4% paraformaldehyde prepared in 0.1 M phosphate-buffered saline at 12 weeks after transplantation. Dissected spinal cords were embedded in Optimal Cutting Temperature compound (Sakura Finetechnical Co., Ltd., Tokyo, Japan) and sectioned in the sagittal/axial

plane at a thickness of 16/20 μm on a cryostat (Leica CM3050 S, Leica Microsystems, Wetzlar, Germany; http://www.leica.com). Sections were histologically evaluated by hematoxylin-eosin staining, LFB staining, and immunohistochemistry. Tissue sections were stained with the following primary antibodies: anti-GFP (rabbit IgG; 1:300; Frontier Institute Co., Ltd., Hokkaido, Japan, Af2020), anti-GFP (goat IgG; 1:200; Rockland Immunochemicals, Gilbertsville, PA, USA, 600-101-215), anti-βIII tubulin (Tuj1; mouse IgG; 1:300; Sigma Chemical Co., St. Louis, MO, USA, T8660), anti-Hu (human IgG; 1:1000; a gift from Dr. Robert Darnell, The Rockefeller University, New York, NY, USA), anti-HNA (mouse IgG; 1:300; Chemicon, Temecula, CA, USA, MAB1281), anti-human cytoplasm (STEM121) (mouse IgG; 1:200; StemCells Inc., Newark, CA, USA, Y40410), anti-APC CC-1 (mouse IgG; 1:200; Abcam, Cambridge, United Kingdom, ab16794), anti-MBP (rat IgG; 1:200; Abcam, ab7349), anti-GFAP (rabbit IgG, 1:200; Dako, Carpinteria, CA, USA, Z0334), anti-human-specific Nestin protein (rabbit IgG; 1:200; Nakamura et al., 2003), anti-NF-H (RT97 monoclonal antibody; mouse IgG; 1:200; Chemicon, MAB5262), anti-5-HT (goat IgG; 1:200; ImmunoStar, Inc., Hudson, WI, USA, 20079), anti-Caspr (mouse IgG; 1:200; Neuromab, CA, USA, 75-001), anti-Kv1.2 (mouse IgG; 1:200; Neuromab, CA, USA, 75-008), anti-Bsn (mouse IgG; 1:200; Stressgen, ADI-VAM-PS003), and anti-hSyn (mouse IgG; 1:200; Chemicon, MAB332). Samples were examined on an inverted fluorescence microscope (BZ 9000; Keyence Co., Osaka, Japan; http://www.keyence.co.jp) or a confocal laser-scanning microscope (LSM 700, Carl Zeiss, Munich, Germany; http://www.zeiss).

Electrophysiology

Electrophysiological experiments were performed using a Neuropack S1 MEB-9402 (Nihon Kohden, Tokyo, Japan; http://www.nihonkohden.co.jp) at 12 weeks after transplantation. Mice were anesthetized with an intraperitoneal

injection of ketamine (60 mg/kg) and xylazine (6 mg/kg), and stimulation was applied through the spinal cord at the occipito-cervical area. For recording of potentials, a needle electrode was placed into a hind limb. The ground electrode was placed subcutaneously. A stimulation with an intensity of 0.8 mA, a duration of 0.2 ms, and an interstimulus interval of 1 Hz was used. The latency was measured as the length of time from the stimulus to the onset of the first wave.

Supplemental References

Nakamura, Y., Yamamoto, M., Oda, E., Yamamoto, A., Kanemura, Y., Hara, M., Suzuki, A., Yamasaki, M., and Okano, H. (2003). Expression of tubulin beta II in neural stem/progenitor cells and radial fibers during human fetal brain development. Laboratory investigation; a journal of technical methods and pathology *83*, 479-489.

Scheff, S.W., Rabchevsky, A.G., Fugaccia, I., Main, J.A., and Lumpp, J.E., Jr. (2003). Experimental modeling of spinal cord injury: characterization of a force-defined injury device. Journal of neurotrauma *20*, 179-193.
