# Supplementary material for: Grafted Human iPS Cell-Derived Oligodendrocyte Precursor Cells Contribute to Robust Remyelination of Demyelinated Axons after Spinal Cord Injury
Source: Stem Cell Reports. 2015 Dec 24;6(1):1–8. doi: 10.1016/j.stemcr.2015.11.013 (PMC4719132; doi:10.1016/j.stemcr.2015.11.013)
Supplement: Document S2. Article plus Supplemental Information [file mmc2.pdf]

Stem Cell Reports
Report
ISSCR
OPEN ACCESS

Grafted Human iPS Cell-Derived Oligodendrocyte Precursor Cells Contribute to Robust Remyelination of Demyelinated Axons after Spinal Cord Injury

Soya Kawabata,[1,2] Morito Takano,[2] Yuko Numasawa-Kuroiwa,[1,3] Go Itakura,[1,2] Yoshiomi Kobayashi,[2] Yuichiro Nishiyama,[1,2] Keiko Sugai,[1,2] Soraya Nishimura,[2] Hiroki Iwai,[2] Miho Isoda,[1] Shinsuke Shibata,[1] Jun Kohyama,[1] Akio Iwanami,[2] Yoshiaki Toyama,[2] Morio Matsumoto,[2] Masaya Nakamura,[2,*] and Hideyuki Okano[1,*]
[1]Department of Physiology, Keio University School of Medicine, 35 Shinanomachi, Shinjuku-ku, Tokyo 160-8582, Japan
[2]Department of Orthopaedic Surgery, Keio University School of Medicine, 35 Shinanomachi, Shinjuku-ku, Tokyo 160-8582, Japan
[3]Department of Pediatrics, Keio University School of Medicine, 35 Shinanomachi, Shinjuku-ku, Tokyo 160-8582, Japan
*Correspondence: hidokano@a2.keio.jp (H.O.), masa@a8.keio.jp (M.N.)
http://dx.doi.org/10.1016/j.stemcr.2015.11.013
This is an open access article under the CC BY license (http://creativecommons.org/licenses/by/4.0/).

SUMMARY

Murine- and human-induced pluripotent stem cell-derived neural stem/progenitor cells (iPSC-NS/PCs) promote functional recovery following transplantation into the injured spinal cord in rodents and primates. Although remyelination of spared demyelinated axons is a critical mechanism in the regeneration of the injured spinal cord, human iPSC-NS/PCs predominantly differentiate into neurons both in vitro and in vivo. We therefore took advantage of our recently developed protocol to obtain human-induced pluripotent stem cell-derived oligodendrocyte precursor cell-enriched neural stem/progenitor cells and report the benefits of transplanting these cells in a spinal cord injury (SCI) model. We describe how this approach contributes to the robust remyelination of demyelinated axons and facilitates functional recovery after SCI.

INTRODUCTION

Previous studies describing functional recovery following transplantation of neural stem/progenitor cells (NS/PCs) in spinal cord injury (SCI) models demonstrated the therapeutic promise of this approach (Cummings et al., 2005; Iwanami et al., 2005). A number of putative underlying mechanisms have been suggested, including cell replacement by grafted NS/PC-derived neurons, astrocytes, and oligodendrocytes; trophic support for increased survival of the host neural cells and host-mediated repair processes; and, more recently, axonal remyelination by grafted NS/PC-derived oligodendrocytes (Cummings et al., 2005; Keirstead et al., 2005; Salewski et al., 2015; Yasuda et al., 2011). Human NS/PCs and human-induced pluripotent stem cell-derived NS/PCs (hiPSC-NS/PCs) predominantly differentiate into neurons, and, to a lesser extent, into mature oligodendrocytes both in vitro and in vivo (Kobayashi et al., 2012; Nori et al., 2011, 2015; Romanyuk et al., 2015). We therefore developed a protocol for the induction of oligodendroglial differentiation of hiPSC-NS/PCs in vitro (Numasawa-Kuroiwa et al., 2014). In the present study, we used a pre-evaluated safe line of induced pluripotent stem cells (iPSCs; 201B7) (Nori et al., 2011, 2015) and induced their differentiation into oligodendrocyte precursor cell-enriched NS/PCs (hiPSC-OPC-enriched NS/PCs). The aim of this study was to evaluate the therapeutic potential of hiPSC-OPC-enriched NS/PCs in the treatment of SCI.

RESULTS

hiPSC-OPC-Enriched NS/PCs Differentiate into Mature Oligodendrocytes In Vitro and In Vivo

We induced hiPSC-OPC-enriched NS/PCs from a pre-evaluated safe iPSC line (201B7) (Nori et al., 2011) following a previously reported protocol (Numasawa-Kuroiwa et al., 2014) (Figure S1). Immunocytochemical analyses revealed that hiPSC-OPC-enriched NS/PCs differentiated into myelin basic protein (MBP)$^+$ mature oligodendrocytes in vitro. This differentiation was not observed using conventional hiPSC-NS/PCs prepared following previously reported protocols (Nori et al., 2011). hiPSC-OPC-enriched NS/PCs also differentiated into β-tubulin isotype III (βIII tubulin)$^+$ neurons and glial fibrillary acidic protein (GFAP)$^+$ astrocytes (Figure 1A). Furthermore, the cytokine expression profile list obtained using a cytokine antibody array showed that significantly higher levels of vascular endothelial growth factor (VEGF) and platelet-derived growth factor (PDGF)-AA were secreted into the culture medium of hiPSC-OPC-enriched NS/PCs than into that of conventional hiPSC-NS/PCs (Figure 1B). Other cytokines such as β-nerve growth factor, brain-derived neurotrophic factor, ciliary neurotrophic factor, glial cell-derived neurotrophic factor, hepatocyte growth factor, neurotrophin-3, and neurotrophin-4 were not detected in either group.

Contusive SCI was induced at the Th10 level in NOD-SCID mice, and 5×10^5 hiPSC-OPC-enriched NS/PCs

CrossMark

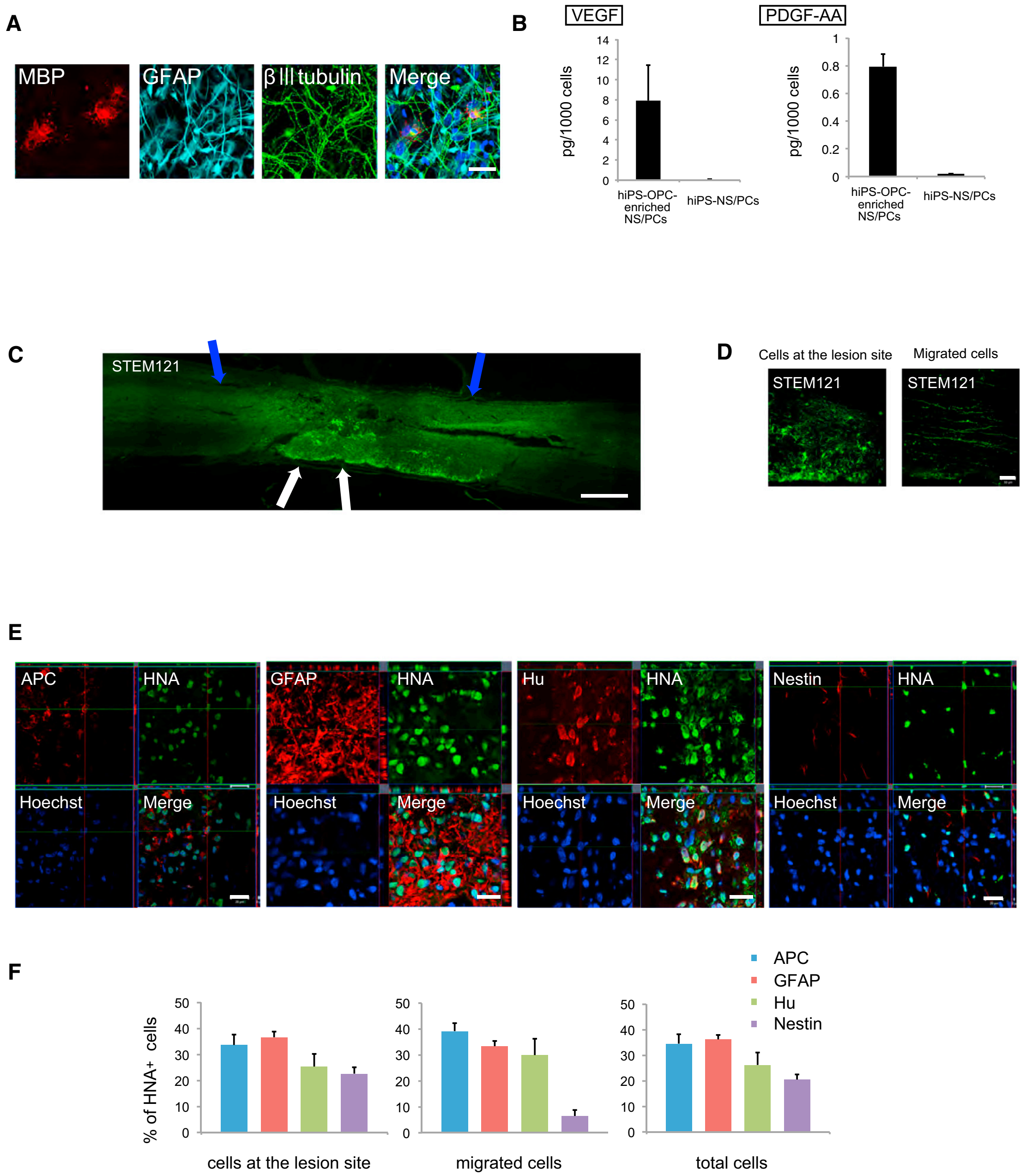

Figure 1. Differentiation Potential of hiPSC-OPC-Enriched NS/PCs In Vitro and In Vivo

(A) Representative immunocytochemical images of three neural lineage cells (neurons, βIII tubulin: astrocytes, GFAP; oligodendrocytes, MBP). Scale bar, 100 μm.

(B) Quantification of secreted cytokines from hiPSC-OPC-enriched NS/PCs and hiPSC-NS/PCs. Secretion of VEGF and PDGF-AA was significantly higher in hiPSC-OPC-enriched NS/PC cultures than in hiPSC-NS/PC cultures (n = 3 independent experiments).

(C) $STEM121^+$ grafted cells were integrated at the lesion epicenter (white arrows) and migrated rostrally and caudally (blue arrows). Scale bar, 500 μm.

(D) Representative images of $STEM121^+$ grafted cells at the lesion site and distant areas. Scale bar, 50 μm.

(E) Representative images of HNA^+ grafted cells together with APC^+ oligodendrocytes, $GFAP^+$ astrocytes, Hu^+ neurons, and $Nestin^+$ immature cells. Scale bar, 20 μm.

(F) Percentages of cell-type-specific marker-positive cells among HNA^+-grafted cells at the lesion site, at distant areas, and in total cells (n = 5 independent experiments).

See also Figures S1 and S2.

were transplanted into the lesion epicenter 9 days later. Twelve weeks after transplantation, we performed immunohistochemical analyses using antibodies specific to human cytoplasm (STEM121) (Cummings et al., 2005), human nuclear antigen (HNA), and cell-type-specific markers. Engrafted hiPSC-OPC-enriched NS/PCs differentiated into adenomatous polyposis coli CC-1 $(APC)^+$ mature oligodendrocytes, Hu^+ neurons, and $GFAP^+$ astrocytes (Figure 1D). Some transplanted cells were present at the lesion site, and the others migrated diffusely into the host spinal cord (Figure 1C). We quantified the proportion of HNA^+ cells immunopositive for cell-type-specific markers in each site. Of the migrated cells, 39.2% ± 3.1% had differentiated into APC^+/HNA^+ mature oligodendrocytes, 33.3% ± 2.0% into $GFAP^+/HNA^+$ astrocytes, 29.9% ± 6.4% into Hu^+/HNA^+ neurons, and 6.34% ± 2.5% into $Nestin^+/HNA^+$ cells. At the lesion site, 33.6% ± 4.1% of HNA^+ cells were APC^+/HNA^+ mature oligodendrocytes, 36.6% ± 2.1% were $GFAP^+/HNA^+$ astrocytes, 25.3% ± 4.8% were Hu^+/HNA^+ neurons, and 22.6% ± 2.5% were $Nestin^+/HNA^+$ cells. Of the total population of differentiated cells within the host spinal cord, 34.5% ± 3.7% were APC^+ cells, 36.1% ± 1.8% were $GFAP^+$ cells, 26.1% ± 5.0% were Hu^+ cells, and 20.4% ± 2.2% were $Nestin^+$ cells (Figure 1E).

Grafted hiPSC-OPC-Derived Mature Oligodendrocytes Contribute to Remyelination

A number of $STEM121^+/MBP^+$ double-positive areas, i.e., areas including human oligodendrocytes, were observed in white matter of the injured spinal cord (Figures 2A–2C), suggesting that many engrafted cells had differentiated into mature oligodendrocytes, forming thick sheathes and migrating diffusely into white matter, but not into gray matter, of the injured spinal cord (Figures 2D–2F). We labeled transplanted cells with Venus fluorescent protein engineered from the original GFP (Nagai et al., 2002). Immunohistochemical analyses revealed large amounts of GFP^+/MBP^+ double-labeled myelin sheaths surrounding NF-H^+ axons (Figure 2G), suggesting that graft-derived human oligodendrocytes form myelin sheaths. Immunoelectron microscopy showed that immature myelin sheaths were strongly associated with myelin cytoplasm with nanogold-labeled GFP^+ spots (Figure 2H). These results indicate that transplanted hiPSC-OPC-derived oligodendrocytes form mature myelin sheaths on spared axons. In addition, nodes of Ranvier, identified with antibodies to the paranodal Caspr protein (Rios et al., 2000) and the juxtaparanodal voltage-gated potassium channel protein Kv1.2 (Wang et al., 1993), were observed in transplanted cell-derived myelin sheaths (Figure 2I). Luxol Fast Blue (LFB) staining showed significant differences between the transplantation and control groups in LFB^+ myelinated areas at all sites examined (Figure 2J). Furthermore, immunohistochemistry revealed that MBP^+ areas in white matter were significantly larger in the transplantation group than in the control group (Figure S3).

Transplanted hiPSC-OPC-Enriched NS/PCs Promote Axonal Growth and Contribute to Synapse Formation between Graft Cell-Derived Neurons and Host Mouse Neurons

We examined the effects of transplanted cells on axonal growth after SCI by immunohistochemical analyses using antibodies for anti-NF-H (a marker of large-diameter neurofilaments) and anti-5-hydroxytryptamine (5-HT; a marker of raphespinal serotonergic fibers). NF-H^+ neuronal fibers were prominent in the transplantation group compared with the control group. There were statistically significant differences in NF-H^+ areas at all sites examined between the two groups (Figure 3A). At the lumbar enlargement, more 5-HT^+ serotonergic fibers were observed in the transplantation group than in the control group (Figure 3B).

To assess the ability of transplanted cell-derived neurons to integrate with the host neural circuitry, we performed triple immunostaining with antibodies to HNA, βIII tubulin, and mouse-specific Bassoon (Bsn), a presynaptic marker. βIII $tubulin^+/HNA^+$ graft-derived neurons co-localized with Bsn^+ synaptic boutons of host neurons (Figure 3C). Triple immunostaining for HNA, βIII tubulin, and human-specific synaptophysin (hSyn) revealed that $hSyn^+$ synaptic boutons were apposed to βIII $tubulin^+/HNA^-$ host mouse neurons (Figure 3C). These findings suggest that graft-derived neurons integrate with host neuronal circuits and form synapses.

Transplanted hiPSC-OPC-Enriched NS/PCs Enhance Functional Recovery Following SCI

Recovery of motor function was assessed by the Basso Mouse Scale (BMS), Rota-rod test, and DigiGait analysis. The BMS score indicated significantly improved motor function in the transplantation group compared with the control group at 35 days post-SCI and beyond (Figure 4A). In the Rota-rod treadmill test, transplanted mice were able to run for significantly longer than control mice at 12 weeks after transplantation (Figure 4B). Using the DigiGait Image Analysis System to analyze gait, we found that stride was significantly longer in the transplantation group than in the control group at 12 weeks after transplantation (Figure 4C).

Because remyelination facilitates the restoration of rapid nerve conduction, we performed electrophysiological examination using the motor-evoked potential (MEP) at 12 weeks post-transplantation. The latency of the MEP in each wave was measured starting from the time of the stimulus. Although we detected an MEP wave in all transplanted mice, we detected MEP waves in only 50% of

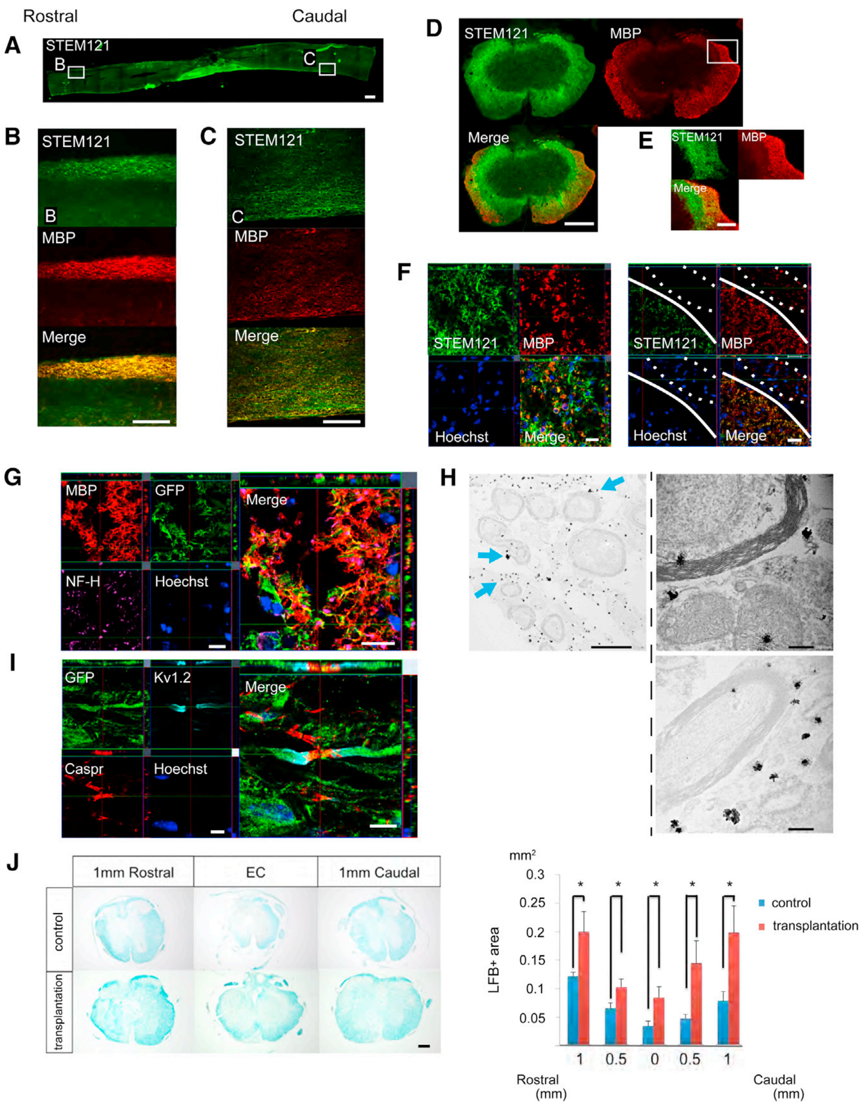

Figure 2. Grafted hiPSC-OPC-Derived Oligodendrocytes Contribute to Remyelination of Spared Axons

(A–C) Representative images of sagittal sections stained for STEM121 and MBP. STEM121$^+$/MBP$^+$ hiPSC-OPC-derived mature oligodendrocytes migrated rostrally and caudally. Scale bar, 500 μm in (A), 300 μm in (B) and (C).

(D–F) Representative images of stained axial sections. hiPSC-OPC-derived oligodendrocytes migrated into white matter broadly. The region enclosed by the dashed line indicates the dorsal root. Scale bar, 300 μm in (D), 100 μm in (E), and 20 μm in (F).

(G) Representative images of axial sections stained for MBP, GFP, and NF-H. Many GFP$^+$/MBP$^+$ double-labeled myelin sheaths were observed around NF-H$^+$ neuronal fibers. Scale bar, 10 μm.

(H) Representative images of immunoelectron microscopy. Grafted cells were detectable by the black dots observed upon anti-GFP antibody staining. Anti-GFP antibody labeling was often observed in the outer cytoplasm of the myelin sheath (blue arrows). At a high magnification, remyelinated axons surrounded by GFP$^+$ transplanted cells were identified. Scale bar, 2 μm (left), 200 nm (right).

(I) Representative images of sagittal sections stained for GFP, Caspr, and Kv1.2. Nodes of Ranvier, identified with antibodies for the paranodal Caspr protein and the juxtaparanodal voltage-gated potassium channel protein Kv1.2, were observed in GFP$^+$ myelin sheathes. Scale bar, 10 μm.

(J) Representative images of LFB staining at the lesion epicenter and at sites 1-mm rostral and caudal. Scale bar, 200 μm. Quantitative analyses revealed a significantly larger myelinated area in the transplantation group than in the control group. Values are means ± SEM (control group, n = 6; transplantation group, n = 5; $^*p < 0.05$).

See also Figure S3.

control mice (Figure 4D). MEP latency was significantly longer in the control group than in the transplantation group (Figure 4D), suggesting that graft-derived oligodendrocytes contribute to an increase in myelinated fibers.

DISCUSSION

Transplanted hiPSC-OPC-Derived Oligodendrocytes Contribute to Remyelination of Spared Axons

The 201B7 hiPSC line is considered to be a safe clone based on previous studies showing the absence of observed tumorigenic propensities upon transplantation into the SCI model of NOD/SCID mice (Nori et al., 2015). In the present study, we used these safe iPSCs and prepared hiPSC-derived OPC-enriched NS/PCs as a cell source to treat SCI in adult NOD-SCID mice. Tumor formation was not observed at 12 weeks after transplantation.

Transplantation of hiPSC-OPC-enriched NS/PCs promoted functional recovery after SCI via differentiation into neurons, astrocytes, and mature oligodendrocytes. Using conventional hiPSC-NS/PCs, approximately 50% of grafted cells differentiate into neurons and only 3% differentiate into APC$^+$ oligodendrocytes (Nori et al., 2011),

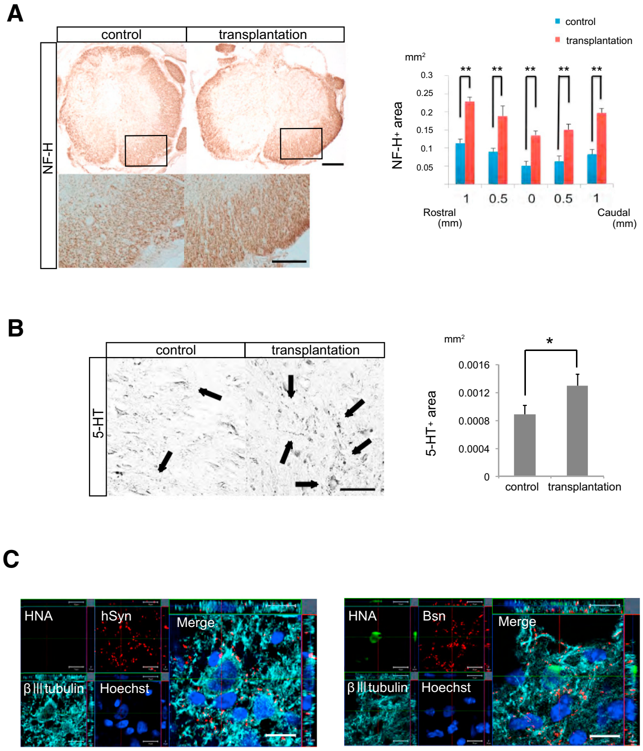

Figure 3. Transplanted hiPSC-OPC-Enriched NS/PCs Promote Axonal Growth and Contribute to Synapse Formation between Grafted Cell-Derived Neurons and Host Mouse Neurons

(A) Representative images of NF-H$^+$ neurofilament staining. Scale bar, 200 μm (upper) and 20 μm (lower). Quantification of NF-H$^+$ areas revealed that neuronal fiber areas were significantly larger in the transplantation group than in the control group. Values are means ± SEM (n = 5, $^{**}p < 0.01$).

(B) Representative images of axial sections stained for 5-HT at 4-mm caudal to the epicenter. Black arrows indicate 5-HT$^+$ fibers. Scale bar, 50 μm. Quantitative analyses revealed that there were significantly more 5-HT$^+$ fibers in the transplantation group than in the control group. Values are means ± SEM (control group, n = 6; transplantation group, n = 5; $^*p < 0.05$).

(C) Representative images showing staining for HNA, βIII tubulin, and the human-specific presynaptic marker hSyn or the mouse presynaptic marker Bsn. hSyn$^+$ boutons were apposed to βIII tubulin$^+$/HNA$^-$ host mouse neurons (left). In addition, βIII tubulin$^+$/HNA$^+$ grafted cell-derived neurons were observed in contact with Bsn$^+$ cells. Scale bar, 10 μm.

while 35% of hiPSC-OPC-enriched NS/PCs differentiate into mature oligodendrocytes. Moreover, immunohistochemistry and electron microscopy revealed direct remyelination of spared axons.

Post-SCI inflammation has both beneficial and deleterious effects on transplanted cells. Inflammation may promote the survival and migration of transplanted cells by inducing the secretion of trophic factors (Copelman

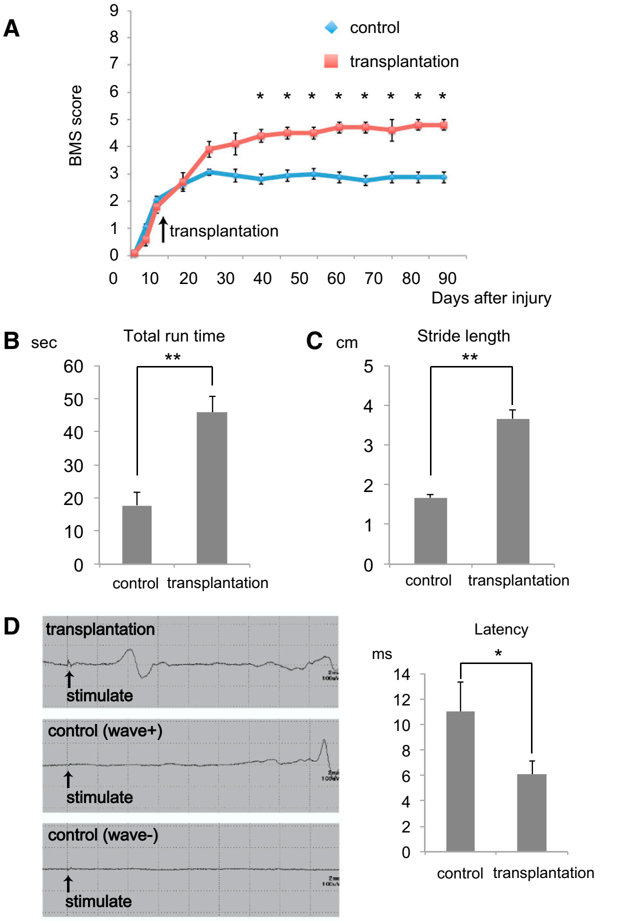

Figure 4. Transplantation of hiPSC-OPC-enriched NS/PCs Promotes Motor Functional and Electrophysiological Recovery after SCI

(A) The BMS score showed that functional recovery was significantly better in the transplantation group than in the control group at 35 days after SCI and thereafter. Values are means ± SEM (control group, n = 8; transplantation group, n = 5; $^*p < 0.05$).

(B) Time on the rotating rod in each group at 12 weeks after transplantation. Transplanted mice could run on the rod for significantly longer than control mice. Values are means ± SEM (control group, n = 8; transplantation group, n = 5; $^{**}p < 0.01$).

(C) Treadmill gait analyses using the DigiGait System at 12 weeks after transplantation. Stride length was significantly longer in the transplantation group than in the control group. Values are means ± SEM (control group, n = 8; transplantation group, n = 5; $^{**}p < 0.01$).

(D) Electrophysiological analyses at 12 weeks after transplantation. Although MEP waves were detected in all transplanted mice, no waves were detected in 50% of control mice. The graph shows the latency of the MEP in transplanted mice and control mice in which waves were observed. The latency was significantly shorter in the transplantation group than in the control group. Values are means ± SEM (control group, n = 5; transplantation group, n = 5; $^*p < 0.05$).

et al., 2000; Diemel et al., 2003; Hinks and Franklin, 1999); however, phagocytosis and the release of pro-inflammatory cytokines by activated macrophages and microglia can harm engrafted cells (Bartnik et al., 2000). One feature of OPCs is their ability to migrate into demyelinated lesions (Piaton et al., 2011). Previous studies using rat models of multiple sclerosis showed that OPCs migrate toward inflamed demyelinated lesions (Wang et al., 2011), suggesting that inflammation affects migration and interactions between OPCs and host tissue. Consistent with this, we found that transplanted hiPSC-OPCs migrate broadly into host white matter and contribute extensively to remyelination of demyelinated axons. Such effects may contribute to the improvement of locomotor function.

Graft Cell-Derived Neurons Contribute to Neuronal Relay by Synapse Formation with Host Mouse Neurons

Previous studies demonstrated post-SCI functional improvement associated with grafted neuronal progenitor cells (Abematsu et al., 2010; Bonner et al., 2010; Cummings et al., 2005). Abematsu et al. (2010) reported that ablation of transplanted iPSC-NS/PCs leads to loss of motor function, suggesting that the survival of graft-derived cells is essential to maintain function. They also showed that graft-derived human neurons receive projections from host mouse neurons, and that their extended processes form synapses with host neurons. In this study, grafted hiPSC-OPC-enriched NS/PCs differentiated into both oligodendrocytes and neurons capable of forming synapses with host neurons within the injured spinal cord, suggesting that the reconstruction of neuronal circuits also plays a substantial role in functional recovery after transplantation of hiPSC-OPC-enriched NS/PCs. All et al. (2015) reported the effectiveness of hiPSC-derived OPC transplantation for SCI in rats. However, their cells predominantly differentiate into oligodendrocytes, and less so into neurons. Graft-derived neurons are also expected to play a significant role in improving functional recovery after transplantation.

We did not transplant conventional hiPSC-NS/PCs in this study because we reported solid data about the transplantation of conventional hiPSC-NS/PCs into an SCI model in NOD/SCID mice (prepared under the same conditions as in the present study) in our previous studies (Nori et al., 2011, 2015). Thus, we simply compared the behavioral data of two groups (animals grafted with hiPSC-OPC-enriched NS/PCs in the present study and those grafted with conventional hiPSC-NS/PCs in previous studies). Although the therapeutic potential of hiPSC-OPC-enriched NS/PCs should in theory differ from that of conventional hiPSC-NS/PCs, we did not find any differences in the BMS score, the Rota-rod treadmill test, or the DigiGait Image Analysis System, which may reflect difficulties in assessing motor function in mice and in differentiating between the two groups. Further studies using larger animal SCI models, such as common marmosets, which will enable us to evaluate skilled movements, may be needed to obtain significant differences in motor function recovery between hiPSC-OPC-enriched NS/PC and hiPSC-NS/PC transplantation.

Grafted Cell-Derived Trophic Factors Enhance the Survival of Host Neural Cells and Host-Mediated Regeneration and Repair without Tumorigenicity

Axonal regrowth supported by astrocytes derived from transplanted cells may be another reason for the observed recovery. A previous report indicated that graft cell-derived immature astrocytes in injured spinal cords promote the outgrowth of 5-HT^{+} fibers by providing a growth-permissive surface (Hofstetter et al., 2002). Consistent with this, transplantation of hiPSC-OPC-enriched NS/PCs promoted serotonergic innervation of the distal cord compared with vehicle control mice, and the release of trophic factors by undifferentiated NS/PCs may protect against neural cell death, and, hence, glial scar formation (Brock et al., 2010; Tuszynski et al., 1996). Here, we show that hiPSC-OPC-enriched NS/PCs secrete several growth factors in vitro, enhance the sparing and/or regrowth of NF-H^{+} neuronal fibers, and prevent atrophy of the injured spinal cord.

In conclusion, OPC-enriched NS/PCs derived from the human iPSC clone 201B7 survived and differentiated into three neural lineage cells in the injured spinal cord of adult mice, with no evidence of tumor formation. Oligodendrocytes derived from transplanted cells may thus play a key role in promoting functional recovery by remyelinating spared axons.

EXPERIMENTAL PROCEDURES

Cell Culture, Neural Induction, and Lentivirus Transduction

Culture and neural induction of hiPSCs (201B7) were performed as described previously (Numasawa-Kuroiwa et al., 2014). Lentivirus was prepared and transduced into neurospheres according to previously described methods (Okada et al., 2008). Briefly, hiPSC-derived primary neurospheres were dissociated and infected with lentivirus-expressing Venus fluorescent protein under the control of the elongation factor promoter (pCSII-EF-Venus). These primary neurospheres were passaged into secondary neurospheres and used for transplantation.

SCI Model and Transplantation

Contusive SCI was induced at the Th10 level in spinal cords of adult female NOD-SCID mice. Nine days after injury, 5×10^{5} hiPSC-OPC-enriched NS/PCs were transplanted into the lesion epicenter of each mouse. All experiments were approved by the

ethics committee of Keio University. Detailed methods are described in the Supplemental Information.

Motor Function Analyses

Hindlimb motor function was evaluated for 12 weeks after transplantation using the BMS (Basso et al., 2006), a rotating rod apparatus, and the DigiGait System (Mouse Specifics). Detailed methods are described in the Supplemental Information.

Histological Analyses

Histological analyses were performed at 12 weeks after transplantation. Sections were evaluated by H&E staining, LFB staining, and immunohistochemistry. Detailed protocols are described in the Supplemental Information.

Statistical Analyses

All data are reported as the mean ± SEM. For all histological examinations, an unpaired two-tailed Student's t-test was used for single comparisons between the transplantation and vehicle control groups. The BMS scores were analyzed using the Mann-Whitney U-test. In each case, $*p < 0.05$ and $**p < 0.01$ were considered to be statistically significant.

SUPPLEMENTAL INFORMATION

Supplemental Information includes Supplemental Experimental Procedures and three figures and can be found with this article online at http://dx.doi.org/10.1016/j.stemcr.2015.11.013.

ACKNOWLEDGMENTS

We thank Prof. Shinya Yamanaka for the human iPSC clones (201B7). This work was supported by grants from the Japan Science and Technology-California Institute for Regenerative Medicine Collaborative Program; Grants-in-Aid for Scientific Research from the Japan Society for the Promotion of Science (SPS) and the Ministry of Education, Culture, Sports, Science, and Technology of Japan (MEXT); and a grant for the Research Center Network for Realization of Regenerative Medicine from the A-MED to H.O. H.O. is a founding scientist and a paid SAB of San Bio, Co., Ltd. The other authors indicated no potential conflicts of interest.

Received: July 26, 2015
Revised: November 25, 2015
Accepted: November 25, 2015
Published: December 24, 2015

REFERENCES

Abematsu, M., Tsujimura, K., Yamano, M., Saito, M., Kohno, K., Kohyama, J., Namihira, M., Komiya, S., and Nakashima, K. (2010). Neurons derived from transplanted neural stem cells restore disrupted neuronal circuitry in a mouse model of spinal cord injury. J. Clin. Invest. *120*, 3255–3266.

All, A.H., Gharibani, P., Gupta, S., Bazley, F.A., Pashai, N., Chou, B.K., Shah, S., Resar, L.M., Cheng, L., Gearhart, J.D., et al. (2015). Early intervention for spinal cord injury with human induced pluripotent stem cells oligodendrocyte progenitors. PLoS One *10*, e0116933.

Bartnik, B.L., Juurlink, B.H., and Devon, R.M. (2000). Macrophages: their myelinotrophic or neurotoxic actions depend upon tissue oxidative stress. Mult. Scler. *6*, 37–42.

Basso, D.M., Fisher, L.C., Anderson, A.J., Jakeman, L.B., McTigue, D.M., and Popovich, P.G. (2006). Basso Mouse Scale for locomotion detects differences in recovery after spinal cord injury in five common mouse strains. J. Neurotrauma *23*, 635–659.

Bonner, J.F., Blesch, A., Neuhuber, B., and Fischer, I. (2010). Promoting directional axon growth from neural progenitors grafted into the injured spinal cord. J. Neurosci. Res. *88*, 1182–1192.

Brock, J.H., Rosenzweig, E.S., Blesch, A., Moseanko, R., Havton, L.A., Edgerton, V.R., and Tuszynski, M.H. (2010). Local and remote growth factor effects after primate spinal cord injury. J. Neurosci. *30*, 9728–9737.

Copelman, C.A., Cuzner, M.L., Groome, N., and Diemel, L.T. (2000). Temporal analysis of growth factor mRNA expression in myelinating rat brain aggregate cultures: increments in CNTF, FGF-2, IGF-I, and PDGF-AA mRNA are induced by antibody-mediated demyelination. Glia *30*, 342–351.

Cummings, B.J., Uchida, N., Tamaki, S.J., Salazar, D.L., Hooshmand, M., Summers, R., Gage, F.H., and Anderson, A.J. (2005). Human neural stem cells differentiate and promote locomotor recovery in spinal cord-injured mice. Proc. Natl. Acad. Sci. USA *102*, 14069–14074.

Diemel, L.T., Jackson, S.J., and Cuzner, M.L. (2003). Role for TGF-beta1, FGF-2 and PDGF-AA in a myelination of CNS aggregate cultures enriched with macrophages. J. Neurosci. Res. *74*, 858–867.

Hinks, G.L., and Franklin, R.J. (1999). Distinctive patterns of PDGF-A, FGF-2, IGF-I, and TGF-beta1 gene expression during remyelination of experimentally-induced spinal cord demyelination. Mol. Cell. Neurosci. *14*, 153–168.

Hofstetter, C.P., Schwarz, E.J., Hess, D., Widenfalk, J., El Manira, A., Prockop, D.J., and Olson, L. (2002). Marrow stromal cells form guiding strands in the injured spinal cord and promote recovery. Proc. Natl. Acad. Sci. USA *99*, 2199–2204.

Iwanami, A., Kaneko, S., Nakamura, M., Kanemura, Y., Mori, H., Kobayashi, S., Yamasaki, M., Momoshima, S., Ishii, H., Ando, K., et al. (2005). Transplantation of human neural stem cells for spinal cord injury in primates. J. Neurosci. Res. *80*, 182–190.

Keirstead, H.S., Nistor, G., Bernal, G., Totoiu, M., Cloutier, F., Sharp, K., and Steward, O. (2005). Human embryonic stem cell-derived oligodendrocyte progenitor cell transplants remyelinate and restore locomotion after spinal cord injury. J. Neurosci. *25*, 4694–4705.

Kobayashi, Y., Okada, Y., Itakura, G., Iwai, H., Nishimura, S., Yasuda, A., Nori, S., Hikishima, K., Konomi, T., Fujiyoshi, K., et al. (2012). Pre-evaluated safe human iPSC-derived neural stem cells promote functional recovery after spinal cord injury in common marmoset without tumorigenicity. PLoS One *7*, e52787.

Nagai, T., Ibata, K., Park, E.S., Kubota, M., Mikoshiba, K., and Miyawaki, A. (2002). A variant of yellow fluorescent protein with fast and efficient maturation for cell-biological applications. Nat. Biotechnol. *20*, 87–90.

Nori, S., Okada, Y., Yasuda, A., Tsuji, O., Takahashi, Y., Kobayashi, Y., Fujiyoshi, K., Koike, M., Uchiyama, Y., Ikeda, E., et al. (2011). Grafted human-induced pluripotent stem-cell-derived neurospheres promote motor functional recovery after spinal cord injury in mice. Proc. Natl. Acad. Sci. USA *108*, 16825–16830.

Nori, S., Okada, Y., Nishimura, S., Sasaki, T., Itakura, G., Kobayashi, Y., Renault-Mihara, F., Shimizu, A., Koya, I., Yoshida, R., et al. (2015). Long-term safety issues of iPSC-based cell therapy in a spinal cord injury model: oncogenic transformation with epithelial-mesenchymal transition. Stem Cell Rep. *4*, 360–373.

Numasawa-Kuroiwa, Y., Okada, Y., Shibata, S., Kishi, N., Akamatsu, W., Shoji, M., Nakanishi, A., Oyama, M., Osaka, H., Inoue, K., et al. (2014). Involvement of ER stress in dysmyelination of Pelizaeus-Merzbacher Disease with PLP1 missense mutations shown by iPSC-derived oligodendrocytes. Stem Cell Rep. *2*, 648–661.

Okada, Y., Matsumoto, A., Shimazaki, T., Enoki, R., Koizumi, A., Ishii, S., Itoyama, Y., Sobue, G., and Okano, H. (2008). Spatiotemporal recapitulation of central nervous system development by murine embryonic stem cell-derived neural stem/progenitor cells. Stem Cells *26*, 3086–3098.

Piaton, G., Aigrot, M.S., Williams, A., Moyon, S., Tepavcevic, V., Moutkine, I., Gras, J., Matho, K.S., Schmitt, A., Soellner, H., et al. (2011). Class 3 semaphorins influence oligodendrocyte precursor recruitment and remyelination in adult central nervous system. Brain *134*, 1156–1167.

Rios, J.C., Melendez-Vasquez, C.V., Einheber, S., Lustig, M., Grumet, M., Hemperly, J., Peles, E., and Salzer, J.L. (2000). Contactin-associated protein (Caspr) and contactin form a complex that is targeted to the paranodal junctions during myelination. J. Neurosci. *20*, 8354–8364.

Romanyuk, N., Amemori, T., Turnovcova, K., Prochazka, P., Onteniente, B., Sykova, E., and Jendelova, P. (2015). Beneficial effect of human induced pluripotent stem cell-derived neural precursors in spinal cord injury repair. Cell Transplant. *24*, 1781–1797.

Salewski, R.P., Mitchell, R.A., Li, L., Shen, C., Milekovskaia, M., Nagy, A., and Fehlings, M.G. (2015). Transplantation of induced pluripotent stem cell-derived neural stem cells mediate functional recovery following thoracic spinal cord injury through remyelination of axons. Stem Cells Transl. Med. *4*, 743–754.

Tuszynski, M.H., Gabriel, K., Gage, F.H., Suhr, S., Meyer, S., and Rosetti, A. (1996). Nerve growth factor delivery by gene transfer induces differential outgrowth of sensory, motor, and noradrenergic neurites after adult spinal cord injury. Exp. Neurol. *137*, 157–173.

Wang, H., Kunkel, D.D., Martin, T.M., Schwartzkroin, P.A., and Tempel, B.L. (1993). Heteromultimeric K+ channels in terminal and juxtaparanodal regions of neurons. Nature *365*, 75–79.

Wang, Y., Piao, J.H., Larsen, E.C., Kondo, Y., and Duncan, I.D. (2011). Migration and remyelination by oligodendrocyte progenitor cells transplanted adjacent to focal areas of spinal cord inflammation. J. Neurosci. Res. *89*, 1737–1746.

Yasuda, A., Tsuji, O., Shibata, S., Nori, S., Takano, M., Kobayashi, Y., Takahashi, Y., Fujiyoshi, K., Hara, C.M., Miyawaki, A., et al. (2011). Significance of remyelination by neural stem/progenitor cells transplanted into the injured spinal cord. Stem Cells *29*, 1983–1994.

Stem Cell Reports, Volume *6*

Supplemental Information

Grafted Human iPS Cell-Derived Oligodendrocyte Precursor Cells Contribute to Robust Remyelination of Demyelinated Axons after Spinal Cord Injury

Soya Kawabata, Morito Takano, Yuko Numasawa-Kuroiwa, Go Itakura, Yoshiomi Kobayashi, Yuichiro Nishiyama, Keiko Sugai, Soraya Nishimura, Hiroki Iwai, Miho Isoda, Shinsuke Shibata, Jun Kohyama, Akio Iwanami, Yoshiaki Toyama, Morio Matsumoto, Masaya Nakamura, and Hideyuki Okano

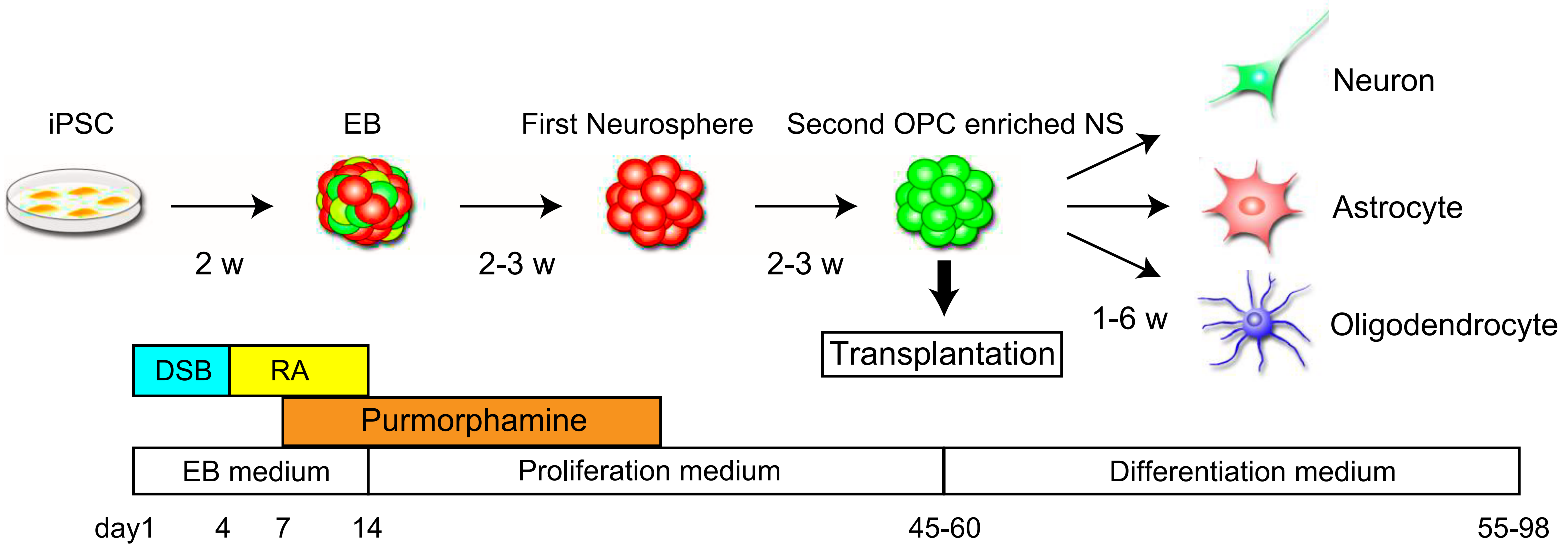
iPSC
EB
First Neurosphere
Second OPC enriched NS
Neuron
Astrocyte
Oligodendrocyte
2 w
2-3 w
2-3 w
1-6 w
Transplantation
DSB
RA
Purmorphamine
EB medium
Proliferation medium
Differentiation medium
day1
4
7
14
45-60
55-98

Figure S1

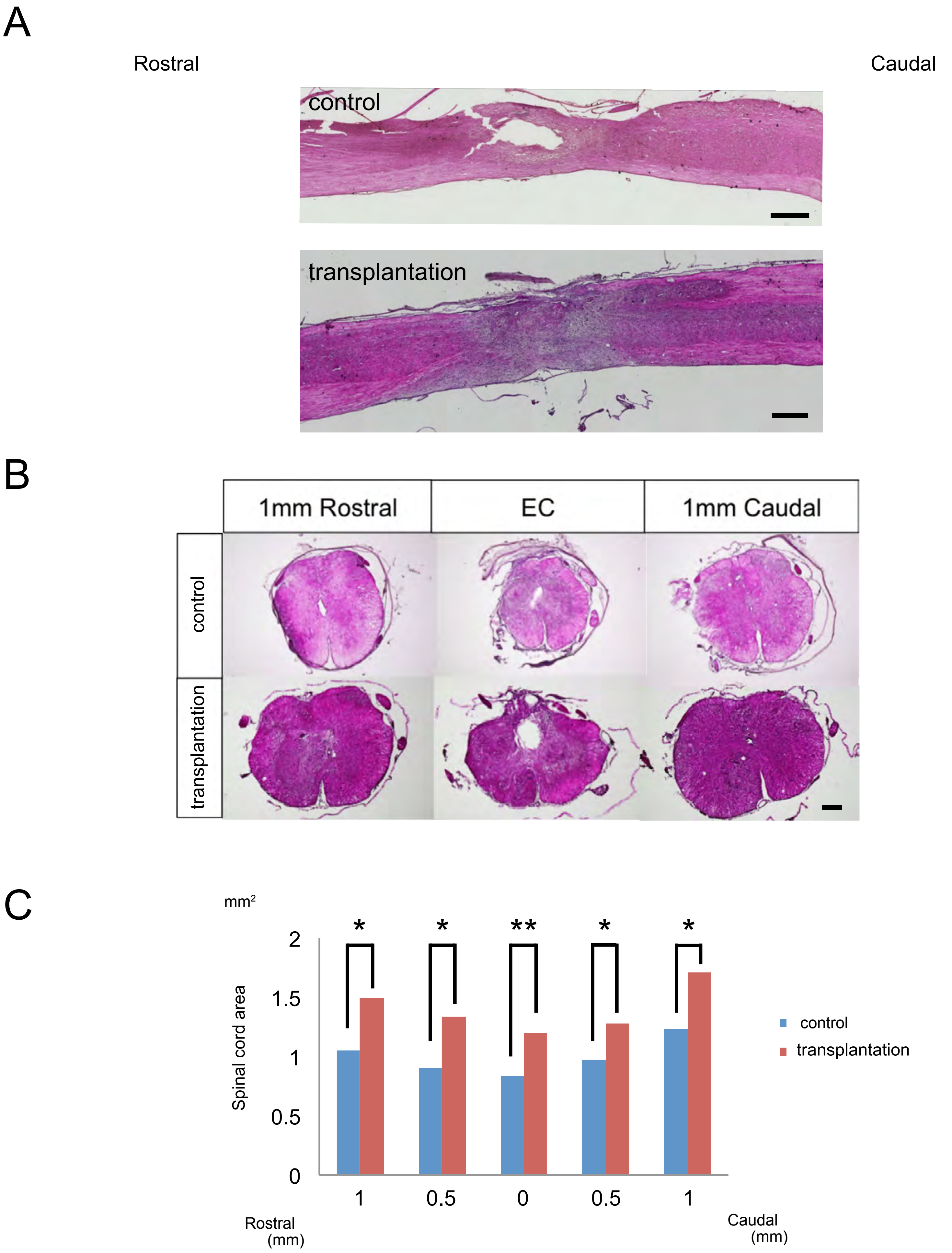
A
Rostral
Caudal
control
transplantation
B
1mm Rostral
EC
1mm Caudal
control
transplantation
C
mm2
Spinal cord area
2
1.5
1
0.5
0
*
*
**
*
*
1
0.5
0
0.5
1
Rostral
(mm)
Caudal
(mm)
control
transplantation

Figure S2

A

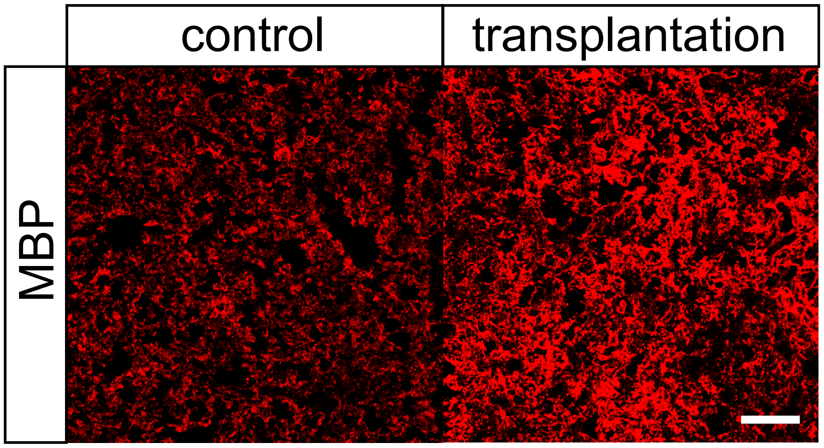
control
transplantation
MBP

B

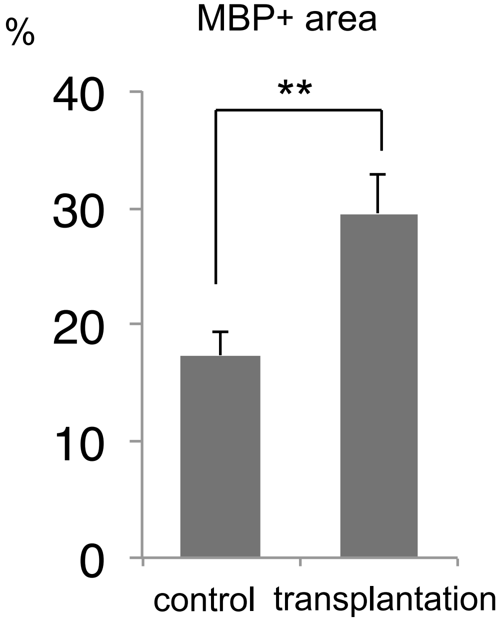
%
MBP+ area
40
30
20
10
0
**
control
transplantation

Figure S3

Supplemental Information

Figure Legends

Figure S1. Differentiation protocols

Schematic presentation of the protocols for hiPSC-OPC-enriched NS/PCs. DSB, dorsomorphin (D), SB431542 (S), and BIO (B); RA, retinoic acid; EB, embryoid body.

Figure S2. Transplantation of hiPSC-OPC-enriched NS/PCs prevents atrophy of the injured spinal cord without any tumorigenicity

A, B. Representative images of hematoxylin-eosin staining of sagittal and axial sections at 12 weeks after transplantation. Tumor formation was not observed in any mice at 12 weeks after transplantation. Scale bar, 500 μm (upper) and 200 μm (lower).

C. Quantitative analysis of spinal cord areas revealed that transverse areas of the injured spinal cord were significantly larger in the transplantation group than in the control group. Values are means ± SEM (control group, n = 6; transplantation group, n = 5; P* < 0.05; P**<0.01).

Figure S3. Transplantation of hiPSC-OPC-enriched NS/PCs enlarges MBP$^+$ areas in white matter

A. Representative images of axial sections stained for MBP. Scale bar, 20 μm.

B. MBP$^+$ percentage areas in white matter. Quantitative analyses revealed that the MBP$^+$ area was significantly larger in the transplantation group than in the control group. Values are means ± SEM (n=5, P** < 0.01).

Supplemental Experimental Procedures

Cytokine array

To obtain conditioned medium, hiPSC-OPC-enriched NS/PCs and conventional NS/PCs were cultured separately in growth medium. At 48 hours before collection, cells were washed once with proliferation medium consisting of serum-free medium and the medium was replaced with fresh growth medium. For background subtraction, the same amount of growth medium without cells was also incubated. The culture media were collected after 48 hours, centrifuged to remove cell debris, and stored at −80°C. The number of viable cells in each culture was calculated for the normalization. Cytokine profiles were obtained using human quantitative custom antibody arrays (RayBiotech, Norcross, GA, USA) to detect nine cytokines, namely, VEGF, PDGF-AA, β-nerve growth factor, brain-derived neurotrophic factor, ciliary neurotrophic factor, glial cell-derived neurotrophic factor, hepatocyte growth factor, neurotrophin-3, and neurotrophin-4, which were measured and processed according to the manufacturer's recommendation. The antibody array was a glass chip-based multiplexed sandwich enzyme-linked immunosorbent assay system. A standard glass slide was spotted with 16 wells of identical biomarker antibody arrays. Each antibody, together with the positive and negative controls, was arrayed in quadruplicate. The samples and standards were added to the wells of the chip array and incubated overnight at 4ºC. This was followed by five washing steps and the addition of a biotinylated antibody and Cy3 equivalent dye labeled-streptavidin to the wells. The signals were scanned and extracted with InnoScan 710 and Mapix software (Innopsys, Carbonne, France). After background subtraction, concentrations were calculated against a standard curve for each biomarker generated from the positive

and negative controls using Quantibody Analyzer software (RayBiotech, Norcross, GA, USA). Cytokine release into culture supernatants was normalized according to the number of viable cells in each culture.

SCI model and transplantation

Adult female NOD-SCID mice (20–22 g) were anesthetized with an intraperitoneal injection of ketamine (100 mg/kg) and xylazine (10 mg/kg). Contusive SCI was induced at the Th10 level using an IH impactor (60 kdyn; Precision Systems and Instrumentation, Lexington, USA), as described previously (Scheff et al., 2003). Nine days after injury, 5×10^5 hiPSC-OPC-enriched NS/PCs were transplanted into the lesion epicenter of each mouse using a glass pipette and a microstereotaxic injection system (KDS310; Muromachi-Kikai Co., Ltd., Tokyo, Japan). An equal volume of phosphate-buffered saline was injected into the lesion site for vehicle control mice.

Motor function analyses

Hind limb motor function was evaluated for 12 weeks after transplantation using the BMS (Basso et al., 2006). Well-trained investigators, blinded to the treatments, performed the behavioral analyses. Motor function was also measured on a rotating rod apparatus (KDS310; Muromachi-Kikai Co., Ltd., Tokyo, Japan), which consisted of a plastic rod (3 cm diameter) with a gritted surface. At 12 weeks after transplantation, each mouse was tested by monitoring the amount of time spent on the rod as it was rotated at 20 rpm. Gait analysis was performed using the DigiGait System (Mouse Specifics, Quincy, MA, USA) at 12 weeks after transplantation.

Histological analyses

All mice were deeply anesthetized and transcardially perfused with 4% paraformaldehyde prepared in 0.1 M phosphate-buffered saline at 12 weeks after transplantation. Dissected spinal cords were embedded in Optimal Cutting Temperature compound (Sakura Finetechnical Co., Ltd., Tokyo, Japan) and sectioned in the sagittal/axial

plane at a thickness of 16/20 μm on a cryostat (Leica CM3050 S, Leica Microsystems, Wetzlar, Germany; http://www.leica.com). Sections were histologically evaluated by hematoxylin-eosin staining, LFB staining, and immunohistochemistry. Tissue sections were stained with the following primary antibodies: anti-GFP (rabbit IgG; 1:300; Frontier Institute Co., Ltd., Hokkaido, Japan, Af2020), anti-GFP (goat IgG; 1:200; Rockland Immunochemicals, Gilbertsville, PA, USA, 600-101-215), anti-βIII tubulin (Tuj1; mouse IgG; 1:300; Sigma Chemical Co., St. Louis, MO, USA, T8660), anti-Hu (human IgG; 1:1000; a gift from Dr. Robert Darnell, The Rockefeller University, New York, NY, USA), anti-HNA (mouse IgG; 1:300; Chemicon, Temecula, CA, USA, MAB1281), anti-human cytoplasm (STEM121) (mouse IgG; 1:200; StemCells Inc., Newark, CA, USA, Y40410), anti-APC CC-1 (mouse IgG; 1:200; Abcam, Cambridge, United Kingdom, ab16794), anti-MBP (rat IgG; 1:200; Abcam, ab7349), anti-GFAP (rabbit IgG, 1:200; Dako, Carpinteria, CA, USA, Z0334), anti-human-specific Nestin protein (rabbit IgG; 1:200; Nakamura et al., 2003), anti-NF-H (RT97 monoclonal antibody; mouse IgG; 1:200; Chemicon, MAB5262), anti-5-HT (goat IgG; 1:200; ImmunoStar, Inc., Hudson, WI, USA, 20079), anti-Caspr (mouse IgG; 1:200; Neuromab, CA, USA, 75-001), anti-Kv1.2 (mouse IgG; 1:200; Neuromab, CA, USA, 75-008), anti-Bsn (mouse IgG; 1:200; Stressgen, ADI-VAM-PS003), and anti-hSyn (mouse IgG; 1:200; Chemicon, MAB332). Samples were examined on an inverted fluorescence microscope (BZ 9000; Keyence Co., Osaka, Japan; http://www.keyence.co.jp) or a confocal laser-scanning microscope (LSM 700, Carl Zeiss, Munich, Germany; http://www.zeiss).

Electrophysiology

Electrophysiological experiments were performed using a Neuropack S1 MEB-9402 (Nihon Kohden, Tokyo, Japan; http://www.nihonkohden.co.jp) at 12 weeks after transplantation. Mice were anesthetized with an intraperitoneal

injection of ketamine (60 mg/kg) and xylazine (6 mg/kg), and stimulation was applied through the spinal cord at the occipito-cervical area. For recording of potentials, a needle electrode was placed into a hind limb. The ground electrode was placed subcutaneously. A stimulation with an intensity of 0.8 mA, a duration of 0.2 ms, and an interstimulus interval of 1 Hz was used. The latency was measured as the length of time from the stimulus to the onset of the first wave.

Supplemental References

Nakamura, Y., Yamamoto, M., Oda, E., Yamamoto, A., Kanemura, Y., Hara, M., Suzuki, A., Yamasaki, M., and Okano, H. (2003). Expression of tubulin beta II in neural stem/progenitor cells and radial fibers during human fetal brain development. Laboratory investigation; a journal of technical methods and pathology *83*, 479-489.

Scheff, S.W., Rabchevsky, A.G., Fugaccia, I., Main, J.A., and Lumpp, J.E., Jr. (2003). Experimental modeling of spinal cord injury: characterization of a force-defined injury device. Journal of neurotrauma *20*, 179-193.
